# Complete Genome Sequence of the Soybean Symbiont *Bradyrhizobium japonicum* Strain USDA6^T^

**DOI:** 10.3390/genes2040763

**Published:** 2011-10-28

**Authors:** Takakazu Kaneko, Hiroko Maita, Hideki Hirakawa, Nobukazu Uchiike, Kiwamu Minamisawa, Akiko Watanabe, Shusei Sato

**Affiliations:** 1 Faculty of Engineering, Kyoto Sangyo University, Motoyama, Kamigamo, Kita-Ku, Kyoto 603-8555, Japan; E-Mail: g0740234@cc.kyoto-su.ac.jp; 2 Faculty of Life Sciences, Kyoto Sangyo University, Motoyama, Kamigamo, Kita-Ku, Kyoto 603-8555, Japan; 3 Kazusa DNA Research Institute, 2-6-7 Kazusa-Kamatari, Kisarazu, Chiba 292-0818, Japan; E-Mails: hmaita@kazusa.or.jp (H.M.); hh@kazusa.or.jp (H.H.); watanabe@kazusa.or.jp (A.W.); ssato@kazusa.or.jp (S.S.); 4 Graduate School of Life Sciences, Tohoku University, Katahira, Aoba-ku, Sendai, Miyagi 980-8577, Japan; E-Mail: kiwamu@ige.tohoku.ac.jp

**Keywords:** genome sequence, symbiosis island, soybean, genomic island, insertion sequence, nodulation, *Bradyrhizobium*, microsymbiont

## Abstract

The complete nucleotide sequence of the genome of the soybean symbiont *Bradyrhizobium japonicum* strain USDA6^T^ was determined. The genome of USDA6^T^ is a single circular chromosome of 9,207,384 bp. The genome size is similar to that of the genome of another soybean symbiont, *B. japonicum* USDA110 (9,105,828 bp). Comparison of the whole-genome sequences of USDA6^T^ and USDA110 showed colinearity of major regions in the two genomes, although a large inversion exists between them. A significantly high level of sequence conservation was detected in three regions on each genome. The gene constitution and nucleotide sequence features in these three regions indicate that they may have been derived from a symbiosis island. An ancestral, large symbiosis island, approximately 860 kb in total size, appears to have been split into these three regions by unknown large-scale genome rearrangements. The two integration events responsible for this appear to have taken place independently, but through comparable mechanisms, in both genomes.

## 1. Introduction

The process of symbiotic nitrogen fixation is of both agricultural and ecological significance. It is a notable component of agricultural production. The establishment of symbiosis between legume plants and rhizobia allows for cooperative functioning between these two organisms, and is vital for agriculture. Soybeans are nodulated by slow-growing bacteria belonging to various species within the genus *Bradyrhizobium* [1]. In general, only some *Bradyrhizobium japonicum* strains are being used in the production of commercial inoculants for soybeans, whereas various other *Bradyrhizobium* spp. span wide regions of the world and exhibit great genetic diversity. Based on geographical and climatic differences in their habitats, the genus *Bradyrhizobium* has maintained a high degree of genetic diversity [2]. For the classification of *B. japonicum*, the strain USDA6^T^ has been used as the type strain for this bacterial species. USDA6^T^ was originally isolated from nodules of a soybean planted in Japan, and has been used for research since it was entered in a culture collection in 1929 [3]. USDA6^T^ has effective symbiotic nitrogen fixation capacity [4].

Since the nucleotide sequence of the genome of *B. japonicum* strain USDA110 was determined in 2002 [5], it has been used for genomic research directed towards investigating soybean symbionts. USDA110 is a strain isolated from the nodule of a soybean grown in Florida, USA in 1957 [4]. Largely because of the superior characteristics of this organism with respect to symbiotic nitrogen fixation, it has been widely used in research in molecular genetics and physiology. The genome of USDA110 is a single circular chromosome, 9.1 Mb in length, and carries 14 genomic islands as DNA segments inserted into tRNA genes [5]. In addition to these genomic islands, a notable large genomic island, known as the symbiosis island, is located downstream of a valine-tRNA gene (*trnV*).

The symbiosis island region carries genes required for the establishment and maintenance of nitrogen-fixing symbiosis with the host plant. The symbiosis island was first identified as an integrated island in the *trnF* gene of *Mesorhizobium loti* ICMP3153 [6]. In addition, the integration of other symbiosis islands into the chromosomes of other rhizobial strains has been reported. It is remarkable that symbiosis islands have only been identified in some rhizobial strains, because, in general, fast-growing rhizobia have the symbiotic plasmid as a cassette for symbiotic nitrogen fixation genes [7]. Complete nucleotide sequences for the islands have been determined for four strains of rhizobia, *M. loti* MAFF303099, *M. loti* R7A, *Azorhizobium caulinodans* ORS571, and *B. japonicum* USDA110 [5,8-10].

The systematic analysis of the gene expression and the structures of these genomes were conducted using DNA array technology methods based on the USDA110 genome sequence [11-15]. Comparative genomic hybridization (CGH), using such DNA array technology, has been applied for the determination of global genome variations among closely related bacteria [16]. CGH analyses of nine strains of *B. japonicum* were performed to clarify their genomic variations [15]. The profiles indicated that the genomes of the nine strains could be classified into three broad genome types (USDA110, USDA122, and USDA6 groups) based on the lower signal intensity from regions located in the putative genomic islands of USDA110.

CGH analysis of *B. japonicum* USDA6^T^ showed genomic variations between USDA6^T^ and USDA110 [15]. Lower intensities were detected in the regions corresponding to all 14 small genomic islands identified on the USDA110 genome, which is a common feature of other members of genomes belonging to the USDA6 group. This observation indicates that the insertions of genomic islands cause obvious variation between USDA110 and the diverse genomes categorized in the USDA6 group. Phylogenetic analyses based on internal transcribed spacer region of ribosomal DNA (ITS) sequence data have also shown that USDA6^T^ belongs to a clade distant from that of USDA110 [15]. These analyses indicate that USDA110 and USDA6^T^ are more divergent than previously thought.

The CGH profile is an effective tool for the categorization of closely related bacterial genomes, but the analysis is restricted to comparisons of regions existing in the reference genome. In order to investigate the genomic features of the type strain of *B. japonicum* and conduct detailed comparative analysis between the two *B. japonicum* strains, we carried out genome sequencing of USDA6^T^, and compared the genomic features between USDA6^T^ and USDA110.

## 2. Results and Discussion

### 2.1. Genome Sequencing

In order to assess the genome size of USDA6^T^ and to confirm its divergence from USDA110 before sequencing, the composition of fragments of genomic DNA digested with two restriction endonucleases, *Pme*I and *Swa*I, was analyzed using pulsed-field gel electrophoresis (PFGE). The sizes of the fragments were estimated by relative mobility analysis and by comparing them to the size markers. The double digestion of USDA6^T^ DNA yielded eight fragments ranging from 95 to 2,200 kb (Table 1). The sum of the fragment sizes was approximately 9.3 Mb. The restriction pattern of USDA6^T^ DNA was different from the predicted pattern based on the USDA110 genome sequence, in terms of fragment number (fifteen fragments) and fragment sizes (from 1 kb to 2,152 kb). This suggests that USDA6^T^ is phylogenetically divergent from USDA110.

**Table 1:** Size of DNA fragments resulting from restriction digestion of the USDA6^T^ genome.

	Total size (kb)	*Pme*I-*Swa*I fragments sizes (kb)
USDA6^T^ (PFGE-gel)	9,295	2,200, 1,850, 1,800, 1,500, 740, 650, 460, 95
USDA6^T^ (computed)	9,207	2,322, 1,702, 1,694, 1,407, 788, 682, 508, 103
USDA110 (computed)	9,106	2,152, 1,911, 1,481, 954, 784, 445, 413, 333, 162, 126, 124, 112, 106, 2, 1

The draft nucleotide sequence of the entire USDA6^T^ genome was deduced initially by assembling a total of 651,020 files containing single-read sequences from the 454/Roche GS-FLX system. The sum of these sequence reads corresponds to 28.7 genome equivalents. The initial assembly produced 151 contigs with lengths of 500 bp or more. The total size of the contigs was 9,195,176 bp.

Two libraries were prepared: a bacterial artificial chromosome (BAC) library (Bj006b) contained inserts of an average length of 82 kb, and a cosmid library (Bj006c) contained inserts of an average length of 30 kb. In total, 2,382 clones from the Bj006b BAC library and 1,929 clones from the Bj006c cosmid library were used to scaffold 137 contigs. The clones were end-sequenced, and these sequences were used to map the contigs, based on output data from BLASTN searches. As a result, these contigs were arranged into a single large scaffold based on the end-sequence information from the Bj006c and Bj006b clones. In order to obtain the nucleotide sequence with accuracy sufficient for subsequent comparative genome analyses, the draft sequences were visually edited and additional sequencing was performed to close the gaps. Integrity assessment of the completed genome sequence was performed by comparing the insert lengths of each Bj006c and Bj006b clone with the computed distance between the corresponding end sequences in the whole-genome sequence.

### 2.2. Structural Features of the USDA6^T^ Genome

#### 2.2.1. Sequence Features

**Figure 1:**
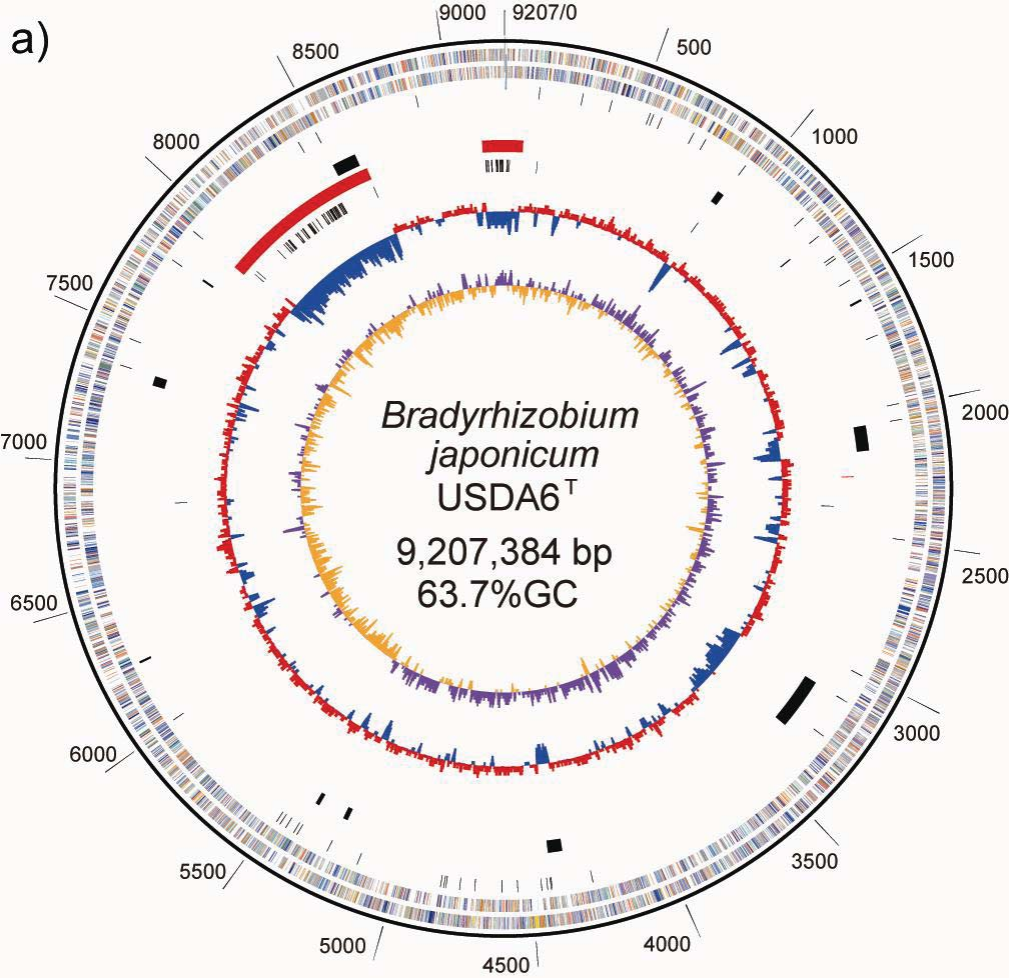

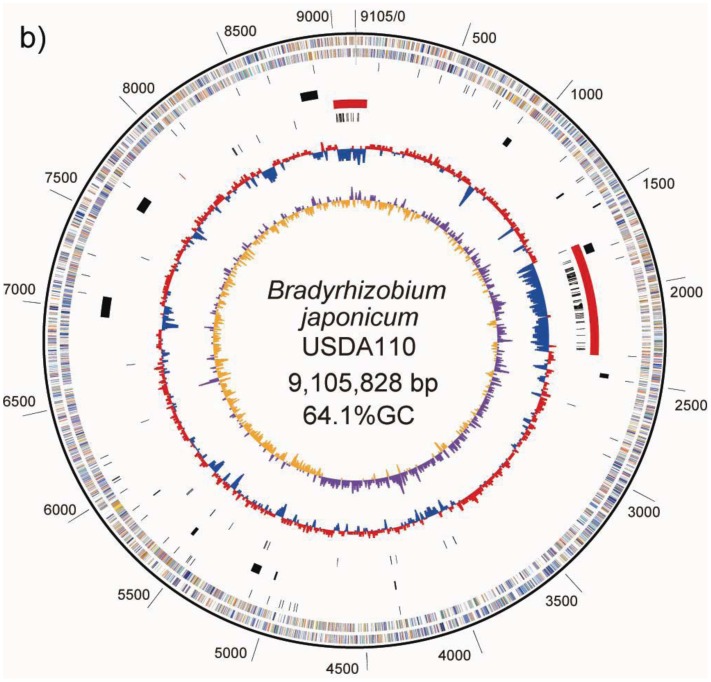
Schematic representations of circular replicons in *B. japonicum* strains. (**a**) USDA6^T^ chromosome and (**b**) USDA110. The scale towards the outside of each map indicates genomic location (in kb). The bars in the outermost circle and the second circle show the positions of the putative protein-encoding genes in clockwise and counter-clockwise directions, respectively. The putative genes are represented by 18 colors, based on Clusters of Orthologous Groups (COG) assignments, as described in Supplemental Figure 1. The third circle from the outside indicates positions of structural RNA genes. In the fourth circle, the black bars indicate areas of putative genomic islands inserted in a tRNA gene. Three red bars within the fifth circle represent regions corresponding to the possible symbiosis island. The sixth circle shows the distribution of insertion sequences (ISs), as black bars. The innermost and second circles from the center show the GC skew values (yellow and purple) and the average GC percentage (blue and red), respectively, calculated using a window-size of 10 kb. This map was depicted using the GenomeViz program.

The chromosome of *B. japonicum* USDA6^T^ is a circular molecule of 9,207,384 bp with an average GC content of 63.67% (Table 2, Figure 1a). No plasmid was detected during the course of this study, including in PFGE patterns. Nucleotide position was assigned as corresponding to the nucleotide sequence initiation site of the *B. japonicum* USDA110 chromosome [5]. Briefly, numbering began at one of the recognition sites of the restriction enzyme *Pac* I (TTAATTAA). Hypothetical PmeI and *Swa*I restriction patterns were calculated for the genome sequence of USDA6^T^, whose size was fixed at 9,207 kb. The restriction patterns are shown in Table 1. The resulting eight fragments, from 103 kb to 2,322 kb, roughly corresponded to the physical restriction patterns of the total DNA seen in PFGE analysis. This supports the structural integrity of the complete genome sequence.

The USDA6^T^ genome sequence is available in public DNA databases (DDBJ/GenBank/EMBL) under the accession number AP012206.

**Table 2:** General features of USDA6^T^ and USDA110 genomes.

	USDA6^T^	USDA 110
Size (bp)	9,207,384	9,105,828
G + C content (%)	63.67	64.06
tRNA coding genes	51	50
rRNA gene clusters	2	1
Protein-encoding genes	8,829	8,317
Gene density (bp)	1,043	1,095
Genes assigned to COG	5,859	5,834
Not in COGs	2,970	2,483
Genomic islands	15	14
Insertion sequences	69	104

#### 2.2.2. rRNA- and tRNA-Encoding Genes

Two copies of the rRNA gene cluster (*rrn*) were identified on the USDA6^T^ chromosome in the order 16S-*trnI-trnA*-23S-5S. One of the *rrns* (*rrn1*) is located at coordinates 1,584,400–1,589,790 bp. The second one (*rrn2*) was found at coordinates 6,251,101–6,256,492 bp, as an exact copy of the first one.

In the case of USDA110, a single copy of *rrn* was identified on the chromosome (Table 2). In the 5′ flanking region of the *rrn* cluster, bll1407 (unknown function) was found, and in the 3′ region of the cluster, bll1408 (unknown function) was found. This gene arrangement (bll1407-*rrn*-bll1408) is conserved in the *rrn1* of USDA6^T^ (BJ6T15130-*rrn1*-BJ6T15190), whereas the organization around *rrn2* is completely different. In the region including *rrn2*, at coordinates 6,233,372-6,260,564, 15 protein-encoding genes, BJ6T61710-BJ6T61810 and BJ6T61870-BJ6T61900, were found, but no orthologs of these genes were found in the USDA110 genome. Briefly, this makes it possible to deduce that the region containing *rrn2* was deleted in the course of evolution of the USDA110 genome.

A total of 51 tRNA genes, including two copies each of *trnI*-GAT and *trnA*-TGC in the *rrns*, which represent 46 tRNA species, are located in the USDA6^T^ genome by prediction analysis using the tRNA scan-SE program. Most of the tRNA genes are dispersed on the genome and are likely to be transcribed as single units, except for those in the rRNA gene cluster. There are four copies of *trnI*-CAU composed of identical sequences (three on USDA110). One copy (BJ6T75390) of the *trnI*-CAU gene is located at 7,790,768–7,790,843 bp and is considered to have originated from the genomic backbone. The other *trnI*-CAU (BJ6T31990) is located within one of the nested genomic islands (BJ6TGI06: described in section “2.2.7. Genomic islands and symbiosis island”) inserted in *trnK*-CUU (BJ6T30200). Two *trnI*-CAUs, BJ6T81540 and BJ6T82220, are encoded by sequences within the symbiosis island, as already shown for the USDA110 genome. The *trnI* gene copies are located within genomic islands, suggesting that they were acquired by lateral gene transfer from an unknown donor.

The nucleotide sequences of the majority of the tRNA genes of USDA6^T^ are identical to or highly similar to (97% identity or more) each of their counterparts in the USDA110 genome. However, two exceptions, *trnS*-GCU (BJ6T46380) and *trnC*-GCA (BJ6T53250), showed lower identities at 84% and 85%, respectively, with their corresponding genes in the USDA110 genome, whereas they showed 97% and 100%, identities, respectively, to their counterparts in *Rhodopseudomonas palustris* CGA009, which belongs to a different clade within the *Bradyrhizobiaceae* [17]. These results show that the USDA6^T^ and USDA110 genomes include intraspecific variations at the molecular level, even in tRNA gene constitution.

#### 2.2.3. Protein-Encoding Genes

Potential protein-encoding regions were predicted using the MetaGeneAnnotator program [18] and a similarity search, as described in section “3.4. Gene assignment, annotation, and information analyses”. The total number of putative protein-encoding genes assigned to the genome was 8,829 (Table 2). The average gene density was estimated to be one gene in every 1,043 bp. Each of the putative protein-encoding genes began with one of three codons: ATG (7,183 genes), GTG (1,084 genes), or TTG (562 genes).

Clusters of Orthologous Groups (COG) assignment for predicted gene products was performed [19]. A total of 5,859 putative gene products encoded by USDA6^T^ were assigned to COG identifications classified into 18 COG categories. For comparison, 5,834 of 8,317 genes in USDA110 were classified into 18 COG categories using the same parameters (Table 2). As shown by comparing the numbers of genes in each category between USDA6^T^ and USDA110 in Table 3, there is a marked underrepresentation of genes classified in category L; “Replication, recombination, and repair” in USDA6^T^. This is ascribed to the presence of a smaller number of transposase genes in USDA6^T^ than in USDA110 [described in 2.2.6. “Insertion sequences (ISs)”].

**Figure 2:**
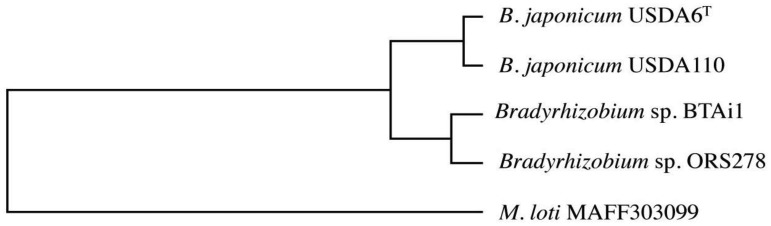
A typical phylogenetic tree using orthologous genes with a single linkage cluster. A gene product (BJ6T39800: recombinase) from USDA6^T^ was selected based on an ortholog cluster corresponding to the phylogenetic pattern of ITS sequences in *Bradyrhizobiaceae*. Orthologs from USDA110 and other *Bradyrhizobium* sp. members (BTAi1 and ORS278) were analyzed using the neighbor-joining method, and the resulting phylogenetic tree is depicted. An ortholog from *M. loti* MAFF303099 was used as the outgroup for this tree.

In order to evaluate conservation of the genes in the genome of USDA6^T^, we carried out ortholog cluster analysis using information on genes predicted in USDA6^T^ and USDA110 as well as in *Bradyrhizobium* sp. BTAi1, *Bradyrhizobium* sp. ORS278, and *Mesorhizobium loti* MAFF303099 [8,20]. The data from this analysis were assessed by focusing on orthologous clusters showing single linkages among the five bacterial strains. As a result, 705 groups were extracted as acceptable orthologous clusters from the grouping data, and 682 of these exhibited patterns corresponding to the phylogenetic tree generated using 16S rRNA genes or ITS sequences from *Bradyrhizobiaceae* members (Figure 2) [15]. This result shows that many of the protein-encoding genes that maintained a single orthologous relationship within *Bradyrhizobiaceae* members had evolved at a similar pace.

**Table 3:** Number of protein genes assigned into Clusters of Orthologous Groups (COG) categories.

COG code	USDA110 entire genome	USDA6^T^	

		entire genome	GIs a
**Information storage and processing**			
J: Translation, ribosomal structure, and biogenesis	197 (2.4%)	203 (2.3%)	1 (0.2%)
K: Transcription	468 (5.6%)	487 (5.5%)	10 (1.7%)
L: Replication, recombination, and repair	333 (4.0%)	290 (3.3%)	30 (5.1%)
**Cellular processes and signaling**			
D: Cell cycle control, cell division, and chromosome partitioning	33 (0.4%)	30 (0.3%)	0 (0.0%)
T: Signal transduction mechanisms	330 (4.0%)	346 (3.9%)	30 (5.1%)
M: Cell wall/membrane/envelope biogenesis	263 (3.2%)	245 (2.8%)	9 (1.5%)
N: Cell motility	243 (2.9%)	233 (2.6%)	9 (1.5%)
O: Posttranslational modification, protein turnover, and chaperones	213 (2.6%)	216 (2.4%)	16 (2.7%)
**Metabolism**			
C: Energy production and conversion	419 (5.0%)	411 (4.7%)	4 (0.7%)
G: Carbohydrate transport and metabolism	389 (4.7%)	409 (4.6%)	4 (0.7%)
E: Amino acid transport and metabolism	717 (8.6%)	710 (8.0%)	14 (2.4%)
F: Nucleotide transport and metabolism	93 (1.1%)	89 (1.0%)	0 (0.0%)
H: Coenzyme transport and metabolism	183 (2.2%)	179 (2.0%)	0 (0.0%)
I: Lipid transport and metabolism	289 (3.5%)	271 (3.1%)	2 (0.3%)
P: Inorganic ion transport and metabolism	281 (3.4%)	296 (3.4%)	5 (0.8%)
Q: Secondary metabolites biosynthesis, transport and catabolism	356 (4.3%)	356 (4.0%)	11 (1.9%)
**Poorly characterized**			
R: General function prediction only	583 (7.0%)	599 (6.8%)	26 (4.4%)
S: Function unknown	444 (5.3%)	489 (5.5%)	19 (3.2%)
not in COGs	2,483 (29.9%)	2,970 (33.6%)	402 (67.9%)
Total	8,317	8,829	592

The percentage of assigned genes out of the total number of genes is shown in parentheses.

a The number of predicted genes assigned inside fifteen GIs of USDA6^T^ is shown.

#### 2.2.4. Putative Replication Origin

A GC-skew analysis was performed to predict the locations of the replication origin and terminus on the chromosome [21]. We observed that shifts in GC-skew values occur in two loci on the USDA6^T^ chromosome, at coordinates 650 and 5,430 kb, as shown in Figure 1a. Shifts of the same sort were found at 705 kb and 4,900 kb in USDA110 (Figure 1b) [5].

The conserved sequence pattern required to convert a dimer chromosome to monomers after aberrant DNA duplication led to the designation of *dif*, a 28-mer sequence [22]. The genome sequence of USDA6^T^ revealed that *dif* is located at coordinates 5,304,527–5,304,554, near one side of the GC-skew shift. It is likely that DNA replication terminates in this region.

Another shift in GC-skew was detected at the diagonal position of *dif*. This seems to indicate the presence of a putative replication origin in the USDA6^T^ chromosome. The occurrence of a gene cluster around this putative origin of replication coincides with other typical origins from the *Bradyrhizobiaceae* [10]. In the case of USDA110, the putative origin is positioned between bll0636 (conserved hypothetical protein) and blr0637 (conserved hypothetical protein). In USDA6^T^, BJ6T06040 and BJ6T06050 were found to be orthologs of these two genes, respectively. This intergenic region showed a nucleotide sequence alignment with 82% identity over 391 bp at coordinates 629,186–629,573 with the corresponding region of the USDA110 sequence. This suggests that the sequence corresponding to the origin of replication is conserved in this region.

#### 2.2.5. Structural Features of the USDA6^T^ Genome

As described in “2.1. Genome Sequencing”, the results of PFGE pattern analysis suggested different genomic proportions, in terms of restriction digest patterns, between USDA6^T^ and USDA110 (Table 1), while the whole-genome sequence analysis focused on sequence conservation between the gene set from USDA6^T^ and the gene set from USDA110. For this purpose, the predicted protein-coding regions of USDA6^T^ were compared with those of USDA110 at the nucleotide sequence level by BLASTN best-hit analysis. The resulting sequence alignments were evaluated based on both the blast bit score of each alignment and the % identity. On this basis, all protein genes were classified into three groups: (1) distinct identical gene, score 100 or more, with over 99% identity; (2) significant similarity, score 100 or more, with less than 99% identity; (3) no similarity, score of less than 100.

**Figure 3:**
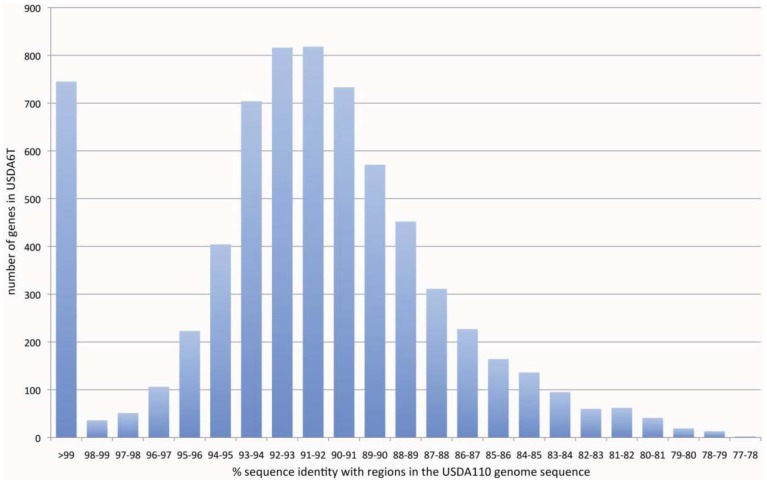
Comparison of USDA6^T^ protein-encoding genes with the USDA110 genome sequence, at the nucleotide sequence level. The plot was generated by using the results of similarity searches of protein-coding regions between USDA6^T^ and USDA110 genome sequences by BLASTN. A total of 6,789 genes with significant similarity were divided into 23 groups based on the % identity in the alignments. The horizontal axis represents the range of % identity in each alignment. The vertical axis represents the number of genes with significant similarity.

As a result of this classification, the number (and percentage) of “distinct identical genes” was 744 (8.5%); the number of genes with “significant similarity” was 6,045 (68.5%); and the number of genes with “no similarity” was 2,039 (23%). Among the USDA6^T^ genes categorized as having “significant similarity”, 3,071 genes have identity percentages within the range of 90% to 94% (Figure 3), and are distributed evenly throughout the genome (data not shown). However, 732 of the genes classified as “distinct identical gene” were found to be limited to three loci with the following coordinates: 2,248,280–2,252,640 (Locus B), 7,920,139–8,614,416 (Locus A), and 9,113,996-0-70,356 (Locus C). The distribution breakdown for the distinct identical genes is: Locus B, seven genes; Locus A, 541 genes; and Locus C, 184 genes. The gene list is shown in Supplemental table 1. These three loci correspond to three parts of the putative symbiosis island (Figure 1a: described in the section “2.2.7. Genomic islands and symbiosis island”).

A dot plot of a BLASTN comparison of the whole-genome sequences of strains USDA6^T^ and USDA110 is presented in Supplemental Figure 2. BLASTN was performed with default parameters, except that the threshold for the alignment was set to a cutoff E-value of 10^−20^. The results from this comparison showed the following genomic characteristics:

(1) Although the dot plot depicts the colinearity of major regions of the two chromosomes, alignment of a region including the replication origin between 8.7 Mb and 1.5 Mb of the USDA6^T^ chromosome harbors a large inversion, compared with USDA110. The two replication origins were positioned similarly; at 0.69 Mb of USDA110 and 0.63 Mb of USDA6^T^;
(2) Many intensively dotted areas in both the 8.0–8.5 Mb and the 9.1–9.2 Mb regions of the USDA6^T^ genome shows the existence of highly copied insertion sequences inside the putative symbiosis island (Table 4: described in section “2.2.6. Insertion sequences”);
(3) A lack of lines indicates regions of unique DNA sequence in each genome. Major deflections of the segments along either axis indicate insertions of genomic islands;
(4) Red lines corresponding to 7.9–8.6 Mb and 9.1–0.7 Mb on the vertical axis indicate the presence of homologous blocks of sequence. The high level of matching sequence alignment suggests that the two regions share a recent common ancestor that includes the insertion of a symbiosis island. These regions match in Locus A and Locus C, described above.

**Figure 4:**
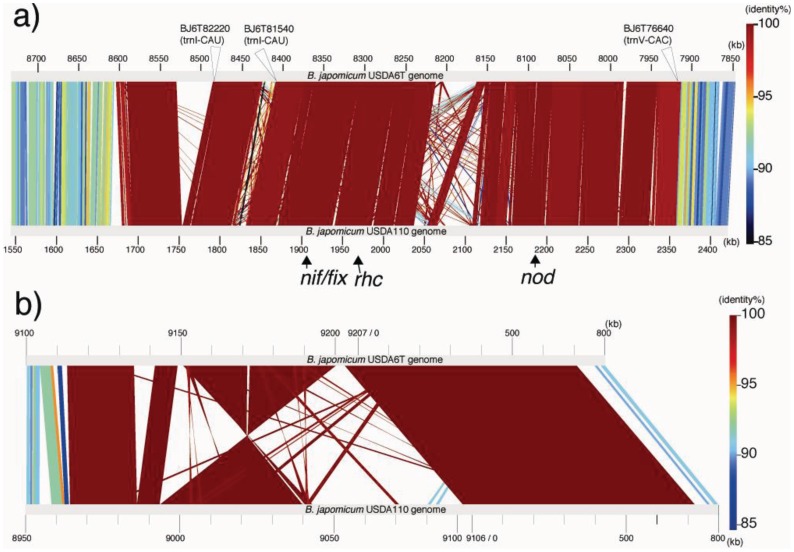
Percent identity plot between USDA110 and USDA6^T^ genome sequences. (**a**) Linear pairwise comparison of regions corresponding to “Locus A” containing “distinct identical genes” from the two genomes. The gray horizontal bars (with a scale corresponding to each genome sequence position), represent the reverse strand of the USDA6^T^ genome and the forward strand of the USDA110 genome. Regions with alignment up to an E-value of 10^−4^ are represented by highlighted connecting colored lines between USDA6^T^ and USDA110. Colors indicate the % nucleotide identity in the alignment output by BLASTN, according to the vertical scale on the right. Open triangles indicate the positions of tRNA genes on the genome. Arrowheads indicate the locations of gene clusters involved in symbiosis or nitrogen fixation; (**b**) Linear pairwise comparison of regions corresponding to the “Locus C”.

Comparisons of linear representations were performed by matching the USDA110 genome with Locus A and Locus C. The approximately 890 kb regions including Locus A are depicted in Figure 4a. The sequence of USDA110 corresponding to Locus A is located at coordinates 1,680,702–2,362,044 of the whole-genome sequence. Locus C is equivalent to a USDA110 region at coordinates 8,974,971-0-70,365 (Figure 4b). The degree of almost all similarities within each long alignment part is represented by red color, denoting their relationships with distinct identical sequences. The fact that these nucleotid sequences in the alignments are included in both coding and intergenic regions shows that there is little intergenic variation in these loci (Figure 4). The variations in these regions depend on the insertion of transposable elements or deletions. Strain-specific islets more than 10 kb in size were found in three parts in USDA6^T^ and four parts in USDA110. Therefore, such events are attributed to the significant variations between these loci.

CGH using a DNA array of USDA110 has been applied to some *B. japonicum* strains, including the USDA6^T^, by Itakura *et al.* [15]. The signal ratio for each probe was plotted along the genome of USDA110. The CGH profile showed that almost all genomic islands of USDA110 were absent in USDA6^T^. However, the signals within the symbiosis island (1.68–2.36 Mb and 8.98-0-0.08 Mb of USDA110 genome) were detected at higher intensity than the ones in the genomic backbone region. Comparative genome analysis shows that the results of the CGH profile analysis indicated the presence of a highly conserved region between the two different strains.

#### 2.2.6. Insertion Sequences (ISs)

ISs are simple mobile DNA elements capable of moving through the genome by transposition. Transposase, an enzyme encoded in ISs, is involved in site-specific DNA recombination required for transposition in bacteria. A number of different ISs found in the bacterial genome sequence have been classified into various families based on structural features of transposases encoded in the ISs [23]. With respect to ISs found in the USDA110 genome, 104 copies have been classified into 20 groups, each comprising 1 to 15 members on the basis of the type of transposase [5]. As shown on the USDA110 map in Figure 1b and Table 4, 62 out of 104 ISs (60%) were located in presumptive symbiosis island A, and 19 (18%) were found in presumptive symbiosis island C (described in the section “2.2.7. Genomic islands and symbiosis island”).

**Table 4:** Distribution of Insertion Sequences (ISs) in the genomes of USDA6^T^ and USDA110.

IS name	USDA110			USDA6^T^		
	
	entire genome	symbiosis island A	symbiosis island C	entire genome	symbiosis island A	symbiosis island C
RSalpha	15	8	2	9	7	2
RSbeta	12	7	4	7	3	4
FK1	6	5	1	5	4	1
IS1632	2	2	0	2	2	0
ISB20	3	2	1	2	1	1
ISB27	3	2	1	3	2	1
ISBj2	10	9	0	10	8	2
ISBj3	4	2	0	1	1	0
ISBj4	6	0	1	1	0	1
ISBj5	5	1	2	5	1	2
ISBj6	1	0	1	0	0	0
ISBj7	11	9	2	10	8	2
ISBj8	4	2	2	5	4	1
ISBj9	2	0	1	0	0	0
ISBj10	1	1	0	2	0	2
ISBj11	5	3	0	1	1	0
ISBj12	1	1	0	2	0	2
ISBj13	3	3	0	1	1	0
ISBj14	7	3	0	1	0	1
ISBj15	3	2	1	2	2	0

Total	104	62	19	69	45	22

To examine the distribution of ISs on the USDA6^T^ genome, similarity searches using the BLASTN program were conducted using the ISs assigned on the USDA110 genome as a query against the entire USDA6^T^ genome sequence. The regions harboring alignments longer than 100 bp, which showed cutoff E-values of up to 10^−60^, were then evaluated. The sequences of these regions were classified according to the corresponding IS groups reported in USDA110. As a result, a total of 69 copies of ISs were assigned identities in the USDA6^T^ genome (Table 4). For all ISs in the USDA110 genome, except ISBj6 and ISBj9, corresponding copies were identified on the USDA6^T^ genome. However, a clear difference in the total copy number of ISs between USDA6^T^ and USDA110 was revealed. This is concordant with the results of the COG analysis, in which USDA6^T^ showed a lower density of transposase genes in the “L” category: Replication, recombination and repair (Table 3).

As shown on the USDA6^T^ map in Figure 1a and Table 4, 45 out of 69 ISs (65%) are located in presumptive symbiosis island A, and 22 (32%) are found in island C (described in the section “2.2.7. Genomic islands and symbiosis island”). Compared with USDA110, there is a marked tendency for ISs in USDA6^T^ to be concentrated in discrete parts of the genome equivalent to the symbiosis island rather than to be distributed evenly throughout the genome (Figure 4a). This observation suggests that there is a comparatively lower degree of plasticity in the USDA6^T^ genome than in the USDA110 genome, due to the smaller number of mobile repetitive elements.

#### 2.2.7. Genomic Islands and Symbiosis Island

A genomic island (GI) is a region that forms syntenic groups of multiple accessory genes encoding diverse functions and characteristics [24]. Acquisition of such a genetic element confers advantages on the bacterium in comparison to other bacteria that colonize the same environment under the same ambient conditions. The majority of GIs occur as traits of integrative elements inserted into the sequenced genome through horizontal transfer. Their sequences are often inserted into various tRNA genes in various bacterial genomes, with the entire tRNA gene flanking the inserted sequence at one end, and the duplicated portion of tRNA gene flanking the inserted sequence at the other end [24].

Duplication of portions of tRNA genes were found at ten loci in the USDA6^T^ genome (Figure 1a). In seven out of ten loci, GIs were identified as a single pair of tRNA genes and the portion. However, multiple duplications of such portion of the tRNA genes were observed in the remaining three loci; four copies in *trnK*-CTT (BJ6T30200), two in *trnQ*-TTG (BJ6T71420), and two in *trnI*-CAT (BJ6T82220) (Table 5). This suggests that discrete GIs were independently integrated into the same tRNA gene, and that this frequently occurred in the course of recent bacterial evolution. Multiple insertions of GIs into *trnK*-CTT and *trnI*-CAT have also been reported in the USDA110 genome [5]. These two tRNA genes may be hotspots of GI integration in *B. japonicum*.

As a result, fifteen varieties of typical GIs with duplicated portions of tRNA genes at both ends were found in the USDA6^T^ genome. The GC content in all islands is lower than the average GC content (63.67%) in the genome (Table 5). The sizes of these GIs are diverse. GI segments ranging from 2.9 to 100 kb separate these duplications, and besides, no apparent nucleotide sequence similarity was detected in any of the fifteen GIs, nor was any such similarity detected in the fourteen GIs from USDA110, except for one pair: BJ6TGI15 in USDA6^T^ and *trnI1*-element in USDA110, which are distinct identical sequences inserted in each symbiosis island.

**Table 5:** List of genomic islands of USDA6^T^.

GI name	tRNA gene (gene_ID)	GI-left end	GI-right end	GI-length (bp)	GC%	duplication length (bp)	gene_ID of integrase
BJ6TGI01	*trnR*-CCG (BJ6T08940)	911,924	937,940	26,017	55.7	50	BJ6T08690
BJ6TGI02	*trnR*-ACG (BJ6T12260)	1,307,833	1,310,731	2,899	62.7	18	-
BJ6TGI03	*trnE*-CTC (BJ6T19770)	2,046,607	2,146,757	100,151	59.7	48	BJ6T20830
BJ6TGI04	*trnK*-CTT (BJ6T30200)	3,118,227	3,192,248	74,022	59.5	46	BJ6T31070
BJ6TGI05	*trnK*-CTT (BJ6T30200)	3,192,295	3,236,376	44,082	60.9	46	BJ6T31620
BJ6TGI06	*trnK*-CTT (BJ6T30200)	3,236,423	3,322,375	85,953	59.0	46	-
BJ6TGI07	*trnK*-CTT (BJ6T30200)	3,322,421	3,326,439	4,019	58.6	45	BJ6T32590
BJ6TGI08	*trnT*-TGT (BJ6T43300)	4,371,641	4,428,687	57,047	58.8	47	BJ6T42570
BJ6TGI09	*trnS*-TGA (BJ6T51900)	5,251,688	5,269,566	17,879	58.4	14	BJ6T51650
BJ6TGI10	*trnN*-GTT (BJ6T53120)	5,380,012	5,394,916	14,905	59.3	53	BJ6T52900
BJ6TGI11	*trnQ*-TTG (BJ6T71420)	7,326,115	7,332,736	6,622	62.7	47	-
BJ6TGI12	*trnQ*-TTG (BJ6T71420)	7,332,777	7,363,200	30,424	62.1	39	-
BJ6TGI13	*trnI*-CAT (BJ6T75390)	7,790,844	7,797,321	6,478	61.1	48	-
BJ6TGI14	*trnI*-CAT (BJ6T82220)	8,494,412	8,539,486	45,075	58.5	49	BJ6T82530
BJ6TGI15	*trnI*-CAT (BJ6T82220)	8,539,534	8,591,258	51,725	59.5	47	BJ6T82650

A total of 592 genes were predicted in the fifteen genomic islands, and 190 of these were assigned into 18 COG categories along with the other genes in the USDA6^T^ genome as described in the section “2.2.3. Protein-encoding genes” (Table 3). This result shows that there is marked overrepresentation of COG categories L (DNA replication, recombination and repair) and T (Signal transduction mechanisms) inside the GIs, due to the presence of integrase and two-component regulator system proteins, respectively. Each of the ten GIs contains a putative integrase gene (Table 5), which implies a function for the GI in integrative events. Although the effects stemming from the addition of two-component regulator system-related genes to the USDA6^T^ genome remain unclear, these genes may confer novel abilities that permit environmental adaptation by USDA6^T^.

There are other potential areas for presumptive GIs within flanking tRNA genes. These regions have no duplications in either end of their segments; however, their sequences show lower GC content. We found five such candidate GIs, with coordinates at 262–288 kb (59.5% GC) in *trnS*-GGA (BJ6T02870), 1,473–1,503 kb (59.6% GC) in *trnL*-CAA (BJ6T14350), 2,488–2,544 kb (61.1% GC) in *trnM*-CAT (BJ6T24130), 8,831–8,882 kb (62.7% GC) in *trnM*-CAT (BJ6T85680) and 7,920–8,614 kb (59.0% GC) in *trnV*-CAC (BJ6T76640). Although each of the first four regions listed above represents a specific sequence in USDA6^T^ compared to USDA110, the putative GI flanking *trnV* is consistent with a region described as Locus A in “2.2.5. Structural features of the USDA6^T^ genome” (Figure 4a), and shows the significant similarity to “symbiosis island A” from USDA110 (Figure 1b) [5].

A symbiosis island carries genes required for nitrogen-fixing symbiosis within the host plant. In the case of USDA110, a 680-kb DNA segment was found to correspond to a symbiosis island [5]. A *trnV*-CAC was found at one end of this region, but there is no trace of any partial terminal duplication of the gene sequence at the other end of this low GC region. However, a 45-bp segment of the 3′ terminal portion of the *trnV* gene flanking 6 kb of segments with lower GC contents was found 3,528 kb away from the *trnV* flanking the symbiosis island (Figure 1b). Another important characteristic of ISs is that they tend to be concentrated in the symbiotic region of the rhizobial genome [7]. The 680-kb segment tagged as a symbiosis island contains 60% (62) of the identified ISs in the genome of USDA110 (Table 4). The other remarkable region containing a high number (19) of ISs and showing low GC content is located at coordinates 8,975-0-70 kb (Figure 1b). Because this 201-kb DNA segment is similar to a symbiosis island in terms of the above features, it is supposed that this segment originated from a common, ancestral, laterally transferred DNA.

A fairly similar situation to USDA110 was found in the USDA6^T^ genome. For USDA6^T^, the presence of a 695-kb DNA region with low GC content at coordinates 7,920–8,614 kb (59.0%) was revealed, with remarkable features reminiscent of a symbiosis island (Figure 1a). This region included most of the *nod*, *nif*, and *fix* genes, and *trnV*-CAC (BJ6T76640) was found at one end of this region at coordinates 7,919,767–7,919,841, but no trace of terminal duplication of *trnV* was detected at the other end of this region. However, a 45-bp segment of the 3′ terminal portion of the *trnV*-CAC gene was found at coordinates 2,248,279–2,248,223, which is 3,536-kb away from the intact *trnV*-CAC gene (Figure 1a), and a putative integrase gene (BJ6T21770) was found next to this tRNA partial segment. The GC content of the 4-kb region (coordinates 2,248–2,252 kb) adjacent to this gene is lower than the average GC content of the entire genome (60.1% *vs.* 63.67%). Among the 69 ISs identified in the USDA6^T^ genome, 45 (65%) are located in the 695-kb region, and 22 (32%) of the remaining ISs are located in the third 164-kb region at coordinates 9,114-0-70 kb, which also has a lower than average GC content (59.85%) (Figure 1a, Table 5). The nucleotide sequences of these three regions correspond to the regions described as Locus A, Locus B, and Locus C in “2.2.5. Structural features of the USDA6^T^ genome”, except for the intervening strain-specific insertions and small inversions (Figure 4b). This suggests that an ancient larger symbiosis island may have been split into three smaller ones: the 695-kb DNA region (putative symbiosis island A at coordinates 7,920–8,614 kb), the 4-kb region (putative symbiosis island B at coordinates 2,248–2,252 kb), and the 164-kb region (putative symbiosis island C at coordinates 9,114-0-70 kb). This is postulated to have taken place by unknown large-scale genome rearrangement through comparable mechanisms after independent integration events into both chromosomes.

### 2.3. Comparative Aspects of Genetic Organization between USDA6^T^ and USDA110

A comparison between the putative gene products of USDA6^T^ and USDA110 was carried out by BLASTP analysis with the aim to determining the evolutionary diversity of *B. japonicum* strains. This analysis revealed that 7,651 (86.7%) putative protein genes in the USDA6^T^ are conserved in the USDA110 genome. Among these conserved genes, 739 genes (8.4% of total genes) show significant high similarity to those in the USDA110 genome (more than 99% identity). Most of the conserved genes with high similarity are located in the symbiosis island. This result is consistent with the nucleotide sequence level comparison as described in “2.2.5. Structural features of the USDA6^T^ genome”.

**Figure 5:**
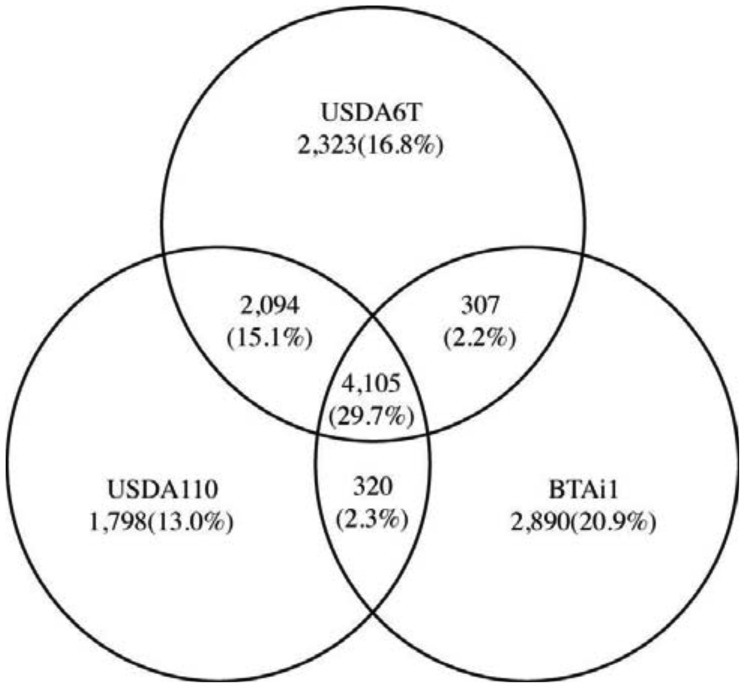
Comparative genomic analysis among three strains: USDA6^T^, USDA110, and BTAi1. The total non-redundant number of deduced proteins from the three strains is 13,837. The number of proteins per genome is given inside the circles representing the bacterial strains. The overlapping sections indicate shared numbers of proteins. The proportion of the entire protein number is shown in parenthesis.

We subsequently performed comparative analysis by comparing genetic data from *Bradyrhizobium* sp. BTAi1 with that from the two *B. japonicum* strains in order to identify the genetic characteristics that are commonly shared by two *B. japonicum* strains. *Bradyrhizobium* sp. BTAi1 possesses the ability to form nodules on stems of *Aeschynomene* spp., and to perform nitrogen fixation in nodules, but BTAi1 has neither symbiosis islands nor symbiotic plasmids [25]. A comparison of the gene constitutions of these strains revealed 4,105 genes commonly conserved in all three strains. This is 29.7% of the combined non-redundant set of genes (13,837) (Figure 5). This group is anticipated to contain the essential genes that are indispensable to support bacterial cellular life, in addition to genes involved in nitrogen fixation and interactions with the host. The number of genes conserved between USDA6^T^ and USDA110 is 2,094 (15.1%). This is significantly larger than 307 (2.2%) between USDA6^T^ and BTAi1 and 320 (2.3%) between USDA110 and BTAi1. These may indicate that many of genes conserved between USDA6^T^ and USDA110 can become the significant candidates in charge of functions involved in soybean symbiosis.

Conserved genes related to nitrogen fixation are present in all three strains. The *nif/fix* gene clusters, *nifDKENX-nifST-nifB-nifZ-nifHQW-fixBCX* and *fixR-nifA-fixA*, are positioned at two loci in the symbiosis islands of both USDA110 (blr1743-bsr1775 and blr2036-blr2038) and USDA6^T^ (BJ6T80440-80760 and BJ6T78160-78180), with identical gene arrangements. Although the BTAi1 genome possesses no symbiosis island, similar arrangements of the *nif/fix* gene clusters (BBta_5869-5923 and BBta_5933-5935) are conserved in the genome, except for *fixA* (BBta_5872) flanking *fixB* (BBta_5871) in the first cluster, which is separated from the second cluster which included *fixR* (BBta_5935) and *nifA* (BBta_5933). Another gene cluster of *fix* genes (*fixK2*, *fixLJ*, *fixNOPQ*, *fixGHIS*), which is essential for microoxic respiration, is positioned outside of the symbiosis island in both USDA110 and USDA6^T^. This cluster is also conserved among all three strains, USDA110 (bll2757-bsr2770), USDA6^T^ (BJ6T70170-70040), and BTAi1 (BBta_2786-2798).

Approximately half of the genes assigned to the symbiosis island were classified into the orthologous group, whose members are conserved between USDA6^T^ and USDA110 (data not shown). The *nod* genes, involved in nodulation, and the *rhc* genes, which encode the type III secretion system, were classified into this group. The *nod* genes *nolZY-nolA-nodD2-nodD1YABCSUIJ-nolMNO-nodZ* are conserved as a single cluster in each strain (bsl2015-blr2035 in USDA110 and BJ6T78190-BJ6T78390 in USDA6^T^), and the *rhc* genes (*ttsI-rhcC2, nopB-rhcJ-nolUV-rhcNQRSTU, rhcC1-nopLC, rhcV;* bll1800-bll1843 in USDA110 and BJ6T80240-BJ6T79820 in USDA6^T^) were also positioned at a single locus within the symbiosis island in each strain (Figure 4a) [26].

*B. japonicum* possesses the capacity to infect plant cells, suggesting that the carbohydrate configuration of the cell surface is important for association with the host during infection [27]. The *B. japonicum* cell is studded with cyclic beta-glucan, lipopolysaccharide (LPS), and exopolysaccharide (EPS) as major extracellular polysaccharides. Some of the genes responsible for synthesis of these polysaccharides and for their secretion have been identified in *B. japonicum* USDA110. The genes for synthesis of cyclic beta-glucan and LPS are conserved in all three of the strains, including *Bradyrhizobium* sp. BTAi1, and are found in a similar arrangement in each gene cluster. However, most of the genes involved in EPS synthesis were classified into the group of genes that are conserved between USDA6^T^ and USDA110 but are not found in *Bradyrhizobium* sp. BTAi1.

Cyclic beta-glucans are localized within the periplasmic compartment, or are excreted into the extracellular medium of the cultures. The cell surface cyclic beta-glucans are recognized to provide functions related to the *B. japonicum* infection process [28]. Three genes, *ndvB* (blr4614), *ndvD* (blr4613), and *ndvC* (blr4612), have been identified as functional genes for biosynthesis of cyclic beta-glucans in USDA110. These genes show significant similarity to the predicted genes, *ndvB* (BJ6T50730), *ndvD* (BJ6T50740), and *ndvC* (BJ6T50750), suggesting a general metabolic pathway for cyclic beta-glucans in USDA6^T^ that is assumed to be similar to that in USDA110.

Lipopolysaccharides (LPSs) are glycolipid molecules, which universally constitute the major component of the outer membrane of proteobacteria. Two LPS biosynthesis-related genes, *lspL* homolog (bll5920) and *galE* (blr5931), were identified in one locus on the USDA110 genome [29]. At this locus, there is a gene cluster that included thirteen genes (bll5919-blr5931) with predicted functions in sugar modification. The gene arrangement in the cluster is completely conserved in USDA6^T^ (BJ6T37870-BJ6T37740).

Exopolysaccharides (EPSs) are bacterial homopolymers composed of a single type of sugar or heteropolysaccharides containing different types of sugars. Rhizobial EPSs are synthesized through a series of sequential steps. Two loci including a cluster of genes involved in EPS synthesis were found in USDA110 [29]. The first cluster (bll7571-blr7578) was identified as *exoUM-metA-exoPTB*. In the USDA6^T^ genome, BJ6T17210-BJ6T17140 at coordinates 1,796,656–1,804,352 contain the counterparts to this *exo* gene cluster. The second cluster in the USDA110 genome consists of ten genes (blr2374-blr2383), including *lspL* (UDP-glucuronic acid epimerase) and *ugdH* (UDP-glucose 6-dehydrogenase). In the USDA6^T^ genome, the region harboring BJ6T74640 to BJ6T74510 corresponds to this gene cluster, except for two genes (BJ6T74530 and BJ6T74540), which are inserted between *lspL* (BJ6T74520) and *ugdH* (BJ6T74550). BJ6T74530 and BJ6T74540 are homologs of the nodulation genes *nolK* (GDP-fucose synthetase) and *noeL* (GDP-mannose 4,6-dehydratase), respectively [30,31]. It is not clear at the moment why the *noeL-nolK* genes are inserted within this cluster in the USDA6^T^ genome, and how these gene products contribute to EPS biosynthesis. Further analysis of these points within the specific portion of the gene cluster remains to be performed.

Two reaction systems, denitrification in free-living cells and the uptake of hydrogen produced during nitrogen fixation, have been investigated in *B. japonicum* [32,33]. The genes encoding key functional enzymes in each system form clusters with other, including functionally related genes, *nosRZDFYLX* (bll0314-blr0320) for denitrification and *hupNCUVSLCDFGHIJK*, *hypABFCDE*, and *hoxXA* (bll6949-bll6927) for the uptake hydrogenase have been identified on the USDA110 genome. However, some *B. japonicum* strains, including USDA6^T^, do not possess genes involved in these functions [15]. The availability of genomic information for USDA6^T^ allows us to compare it with the USDA110 genome and characterize its gene constitution, thus providing a glimpse into the source of differences in gene distribution between the two strains. Comparative analysis of these genes showed that *nosRZDFYLX* (bll0314-blr0320) was a unique gene set in USDA110. No functional component of the *nos* gene cluster was found in USDA6^T^, and thus USDA6^T^ would be expected to lack nitrous oxidase activity. Denitrification in *B. japonicum* also depends on the nitrate reductase (*napEDABC:* bsr7036-blr7040), nitrite reductase (*nirK:* blr7089) and nitric oxide reductase (*norCBQD:* blr3214-blr3217) [34], however these USDA6^T^ orthologs were found (BJ6T23740-BJ6T23700, BJ6T23260 and BJ6T65370-BJ6T65340, respectively). Notably, the coding region for the *nos* gene cluster (bll0314-blr0320) in USDA110 flanks the *trnS*-GGA gene at coordinates 333,518–333,607, while the flanking region of *trnS*-GGA (BJ6T02870) in USDA6^T^ suggested a genomic island at 262–288 kb (59.5% GC) as described in “2.2.7 Genomic islands and symbiosis island”. This suggests that the *nos* gene cluster is part of an unknown GI in USDA110 and that it was possibly inserted in the USDA110 genome during the course of evolution.

A complete set of *hup-hyp-hox* cluster genes, *hupNCUVSLCDFGHIJK*, *hypABFCDE* and *hoxXA* (bll6949-bll6927), was found at coordinates 7,624,531–7,649,197 in the USDA110 genome [5]. This indicates that this strain has the functional role of hydrogenase uptake [35]. Since this gene cluster is found inside the genomic island *trnM1*-element at coordinates 7,619,541–7,701,499, it is possible that high nitrogen fixation by USDA110 might have been acquired through the insertion of a GI into its genome. On the other hand, the *hup-hyp-hox* cluster is missing from USDA6^T^. In USDA6^T^, the *trnM*-CAT gene (BJ6T24130), corresponding to the one targeted by the *trnM1*-element of USDA110, was detected. However, the tRNA gene (BJ6T24130) is flanked by a region of lower GC content (61.1% GC) at coordinates 2,488–2,544 kb whose sequence is not coincident with ones of the *trnM1*-element. The existence of such tRNA gene (BJ6T24130) in USDA6^T^ may show that it has the potential to gain the ability to take up the hydrogen and to improve efficiency of nitrogen fixation.

USDA110 possess a *trb* gene cluster, *trbBCDEJLFGI* (bll8279-bll8288). The *trb* genes are related to conjugal transfer of mobilizable genetic elements, and this cluster bore a marked similarity to the *virB* gene cluster, which comprises the Type IV secretion system (T4SS) [36]. Notably, the *trbLFGI* genes showed similarity to *virB6-B10* genes, which encode proteins that comprise the channel for the Type IV secretion system [37]. On the other hand, *trbCDE* genes have similarity to *virB2-B4* genes as well. *virB2* encodes pilus components, VirB3 is an assembly factor for T pilus formation, and *virB4* encodes an ATPase [37]. In the USDA110 genome, *trbBCDEJLFGI* (bll8279-bll8288) was found at coordinates 9,068,090–9,077,553. This locus is partly comprised of a region conserved between USDA110 and USDA6^T^ (Figure 4b). This region corresponds to the “putative symbiosis island C” described in section “2.2.7 Genomic islands and symbiosis island”. However, this gene cluster could not be identified in the USDA6^T^ genome. Presumably, it would have been deleted from the corresponding region in the USDA6^T^ genome. The effect of a lack of *trb* genes is uncertain. If the *trb* genes are functionally related to T4SS, their deletion in USDA6^T^ might cause differences in host range between USDA6^T^ and USDA110.

## 3. Experimental Section

### 3.1. Bacterial Strain and Genomic Libraries

*Bradyrhizobium japonicum* strain USDA6^T^ and its cultivation have been described in Itakura *et al.* [15]. The entire genome of USDA6^T^ was sequenced using Sanger-ABI3730 and 454/Roche GS-FLX machines. Total cellular DNA for 454 sequence analysis was purified according to the instruction manual for QIAGEN Genomic-tip (QIAGEN, Hilden, Germany). After the DNA was sheared by nebulization, a genomic library containing inserts of 0.55 kb in average length was constructed to perform pyrosequencing. For genome sequencing using the Sanger method, two random genomic libraries were constructed from the total cellular DNA of USDA6^T^. Megabase genomic DNA embedded in agarose plugs was used for construction of BAC and cosmid libraries as previously mentioned [38,39]. The BAC library (Bj006b) contained inserts of 82 kb in average length that were cloned into a BAC vector, pCC1BAC (Epicentre Bio., Madison, WI, USA), and the cosmid library (Bj006c) contained inserts of 30 kb in average length that were cloned into the cosmid vector pKS800 [39].

### 3.2. Pulsed-Field Gel Electrophoresis

The DNA fragments were separated by pulsed-field gel electrophoresis (PFGE). Preparation of intact genomic DNA and restriction enzyme digestion of DNA were performed based on the protocol reported previously [40]. In brief, the DNA was digested with two restriction endonucleases, *Pme*I and *Swa*I (NEB, Ipswich, MA, USA). The resulting fragments were resolved using the CHEF Mapper gel electrophoresis system (BioRad, Richmond, CA, USA) with 0.5× TBE buffer and 1% Pulsed field agarose (BioRad) at 14 °C in a temperature controlled cooling unit. To facilitate separation of fragments of 50–1,000 kb in size, contour-clamped homogeneous electric field (CHEF) electrophoresis was conducted using a pulse time of 40–60 seconds, field strength 6 V/cm, and run time of 24 h. Two stepwise CHEF conditions were applied to resolve the larger fragments as follows. The first step utilized a pulse time of 1,200–1,469 seconds, field strength of 2 V/cm, and runtime of 72 h. The second step utilized a pulse time of 79–146 seconds, field strength of 6 V/cm, and runtime of 11 h. *Saccharomyces cerevisiae* chromosomes, *Hansenula wingei* chromosomes (BioRad), and MidRange I PFG Marker (NEB) were used as size standards for the PFGE analyses.

### 3.3. DNA Sequencing

The whole-genome shotgun method was applied to genome sequencing using sequence data obtained by the use of a 454/Roche GS-FLX machine, in combination with BAC end-sequencing. Pyrosequencing for the production of the shotgun single reads was performed using one-half of the picotiter plate in the 454/Roche GS-FLX system, resulting in 28.7-fold (651,020 reads) sequence coverage of the genome. The sequences at both ends of the clones from the Bj006b and Bj006c libraries were analyzed using a Dye-terminator Cycle Sequencing Kit and a 3730XL Sequencer (Applied Biosystems, Foster City, CA, USA). The end-sequence data from the BAC clones facilitated the gap-closure process and provided the scaffolding for reconstruction of the sequence of the entire genome [5].

The accumulated sequence files were assembled in two steps. A total of 651,020 sequence files were processed using a MIRA3 assembler [41]. The contigs resulting from the initial assembly were read out as consensus sequences in FASTA format. The contig sequences were assembled together with the end-sequences from 3,072 BAC and 1,968 cosmid clones using Phred/Phrap software [42]. GSFLX-derived data and Sanger-derived reads for USDA6^T^ were imported into Consed [42], edited, and verified. We filled the remaining gaps in the sequence by primer walking, using the cosmids or the BAC clones as templates. The integrity of the reconstructed genome sequence was assessed by chromosome walking using the end sequences of the BAC clones.

### 3.4. Gene Assignment, Annotation, and Information Analyses

Regions encoding rRNA genes (16S, 23S, and 5S) were identified by similarity searches against data deposited into an in-house database, RhizoBase [43]. tRNA genes were predicted using the tRNA scan-SE 1.23 program [44].

Prediction of protein-coding regions was carried out using a combination of the MetaGeneAnnotator program [18] and similarity searches against predicted genes from the USDA110 genome. All protein-coding regions 120 bp or longer were translated into amino acid sequences. The putative protein-encoding genes used either ATG, GTG, or TTG as their start codons. All of the predicted genes for structural RNAs and proteins were denoted following their ordering on the genome by a serial number with the prefix “BJ6T”, representing the strain USDA6^T^. The putative protein-encoding genes were subjected to subsequent similarity searches against the protein databases. In order to assign functions to the protein-encoding genes, they were annotated based on the best hit information from sequence similarity searches against a gene set derived from the USDA110 genome database.

Comparison between two genomic nucleotide sequences, USDA6^T^ and USDA110, was performed using GenomeMatcher v.1.6 [45]. GC-skew analysis was performed as described by Lobry [46]. To examine the similarity of the nucleotide sequences of the USDA6^T^ genes or genome to those of the USDA110 strain, similarity searches was conducted using the BLASTN program [47].

In order to characterize the protein gene products encoded by USDA6^T^, similarity searches using the BLASTP program were performed against databases including all protein gene products from the USDA110 genome or the *Bradyrhizobium* sp. BTAi1 genome, and against the NCBI non-redundant protein sequences (nr) database [48]. A cutoff E-value of 10^−4^ was considered significant. Predicted protein-encoding genes from USDA6^T^ and USDA110 were classified into the categories of COGs according to the results of BLASTP searches against the COG reference data set [19]. Functional motifs of the predicted gene products in the genomes of USDA6^T^ and USDA110 were assigned using the HMMSCAN program with domain searches against the Pfam 25.0 database [49,50].

Orthologous genes among the three strains, USDA6^T^, USDA110, and *Bradyrhizobium* sp. BTAi1, were predicted by reciprocal BLAST best hit with E-value cutoff of 10^−4^. Orthologous relationships were depicted in a Venn diagram. The genes of five bacterial strains investigated, *B. japonicum* USDA6^T^, USDA110, *Bradyrhizobium* sp. BTAi1, *Bradyrhizobium* sp. ORS278, and *M. loti* MAFF303099, were classified into clusters by single linkage clustering using the BLASTCLUST program using as criteria, 60% or more amino acid identity and the alignment extended over at least 0.7 times the length of the query sequence. Phylogenetic patterns of the orthologous genes were inferred for the amino acid sequences using the neighbor-joining algorithm and the CLUSTALW program [51]. Phylogenetic trees were constructed with MEGA 5 software [52].

## 4. Conclusions

The genome of *B. japonicum* USDA6^T^ was sequenced and the genomic features of two strains, USDA6^T^ and USDA110 were compared. The analysis revealed typical genomic features of the type strain of *B. japonicum*, and integration of a symbiosis island similar to one of USDA110 into the USDA6^T^ genome. The present results suggest that a larger symbiosis island, approximately 860 kb in size, was retained in an ancestral genome. The progenitor symbiosis island was split into three regions by unknown large-scale genome rearrangements through comparable mechanisms after independently integrating into both genomes. The genome information for USDA6^T^, along with that for USDA110, provides new insights into the genetic diversity of the genus *Bradyrhizobium*, which could be used to further study the evolutionary relationships among soybean symbionts as well as their individual characteristics.

## Supplementary Files

Supplementary File 1 PDF-Document (PDF, 917 KB)
