# Supplementary material for: Complete Genome Sequence of the Soybean Symbiont Bradyrhizobium japonicum Strain USDA6T
Source: Genes (Basel). 2011 Oct 28;2(4):763–87. doi: 10.3390/genes2040763 (PMC3927601; doi:10.3390/genes2040763)
Supplement: Supplementary File 1 — PDF-Document (PDF, 917 KB) [file genes-02-00763-s001.pdf]

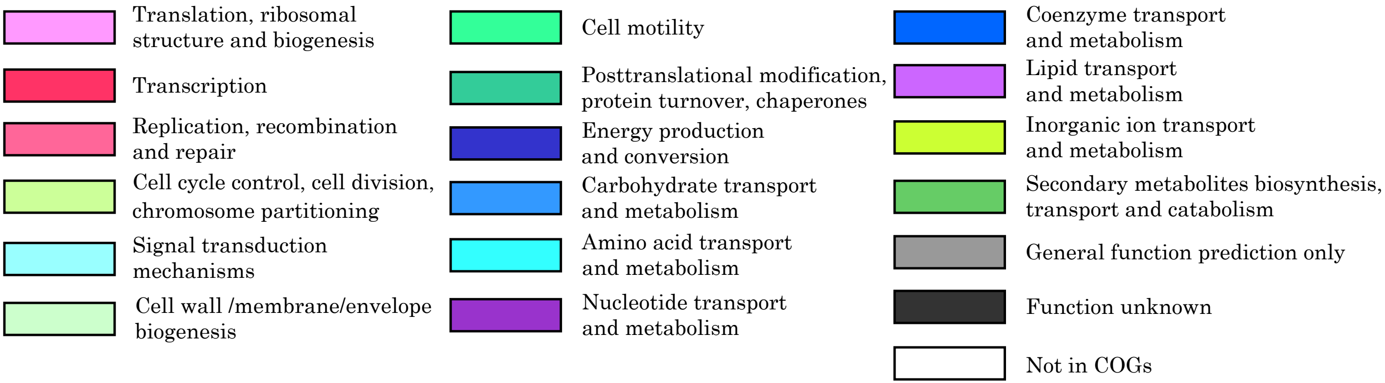


**Supplemental Figure 1.** Color codes for functional categories of the predicted proteins. The bars showing the positions of the putative protein-encoding genes are arranged on two circular representations depicted in Figure 1. The bars are represented by 18 colors, based on Clusters of Orthologous Groups (COG) assignments.

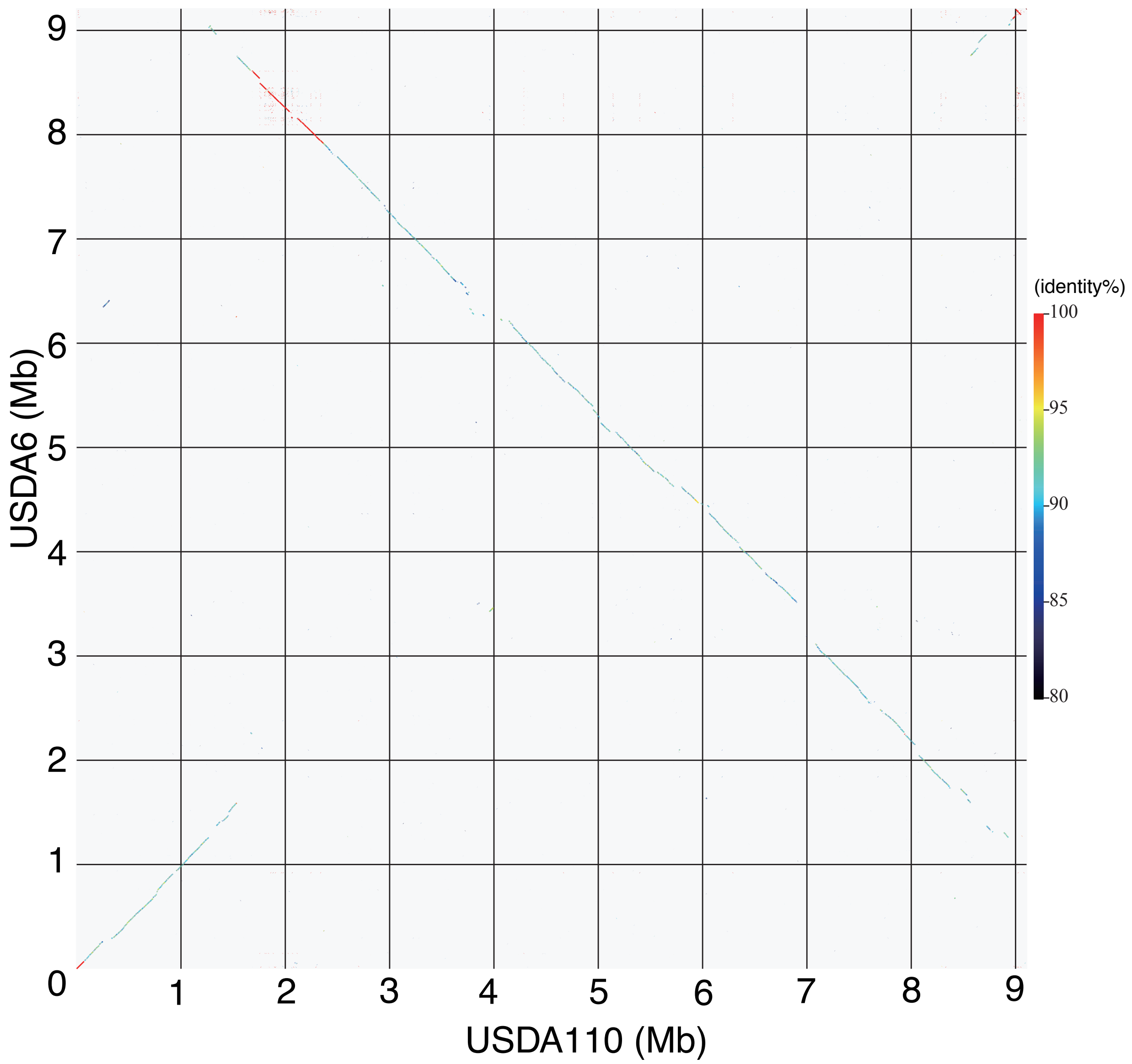


**Supplemental Figure 2. Dot plot of a BLASTN comparison of strains USDA110 and USDA6 whole-genome sequences.** Horizontal axis: position on the USDA110 genome sequence (Mb); vertical axis: position on the USDA6 genome sequence (Mb). Colors indicate the % nucleotide identity in the alignment output by BLASTN, according to the scale on the right.

## Supplemental Table1　List of all predicted genes for each locus A, B, C (putative symbiosis island)

| | gene_ID(locus_tag) | left_position | right_position | direction | COG_category | | product_name | gene_name | USDA110gene |
|---|---|---|---|---|---|---|---|---|---|
| locusB | BJ6T21770 | 2248480 | 2249811 | + | L | COG0582 | probable site-specific integrase/recombinase | | bll7217 |
| locusB | BJ6T21780 | 2249756 | 2250121 | − | | | hypothetical protein | | blr7216 |
| locusB | BJ6T21790 | 2250440 | 2250811 | | | | hypothetical protein | | |
| locusB | BJ6T21800 | 2250774 | 2251244 | + | | | hypothetical protein | | bll7214 |
| locusB | BJ6T21810 | 2251291 | 2251500 | + | | | hypothetical protein | | bll7214 |
| locusB | BJ6T21820 | 2251789 | 2251974 | | | | hypothetical protein | | |
| locusB | BJ6T21830 | 2252197 | 2252640 | + | R | COG0824 | hypothetical protein | | bll7212 |
| | | | | | | | | | |
| locusA | BJ6T76650 | 7920139 | 7921173 | + | | | hypothetical protein | | bll2187 |
| locusA | BJ6T76660 | 7921170 | 7921550 | + | | | hypothetical protein | | bll2186 |
| locusA | BJ6T76670 | 7921602 | 7922600 | + | R | COG1204 | hypothetical protein | | bll2185 |
| locusA | BJ6T76680 | 7922645 | 7923088 | + | | | hypothetical protein | | bll2184 |
| locusA | BJ6T76690 | 7923107 | 7923403 | + | | | hypothetical protein | | bsl2183 |
| locusA | BJ6T76700 | 7924049 | 7924279 | + | | | hypothetical protein | | bsl2182 |
| locusA | BJ6T76710 | 7924332 | 7924493 | + | | | hypothetical protein | | bsl2181 |
| locusA | BJ6T76720 | 7924527 | 7925723 | − | T | COG0642 | hypothetical protein | | blr2180 |
| locusA | BJ6T76730 | 7926093 | 7926326 | + | T | COG0784 | hypothetical protein | | bsl2179 |
| locusA | BJ6T76740 | 7926765 | 7928447 | − | T | COG0642 | two-component hybrid sensor and regulator | | blr2178 |
| locusA | BJ6T76750 | 7928434 | 7929954 | − | T | COG0467 | hypothetical protein | | blr2177 |
| locusA | BJ6T76760 | 7930165 | 7932159 | + | T | COG2202 | two-component hybrid sensor and regulator | | bll2176 |
| locusA | BJ6T76770 | 7932265 | 7932450 | + | | | hypothetical protein | | bsl2175 |
| locusA | BJ6T76780 | 7932667 | 7933611 | − | C | COG1012 | malonic semialdehyde oxidative decarboxylase | *iolD* | blr2174 |
| locusA | BJ6T76790 | 7933658 | 7934128 | + | N | COG3839 | hypothetical protein | | bll2173 |
| locusA | BJ6T76800 | 7934163 | 7935053 | + | G | COG3386 | hypothetical protein | | bll2172 |
| locusA | BJ6T76810 | 7935099 | 7936898 | − | S | COG1593 | probable ABC transporter permease protein | | blr2171 |
| locusA | BJ6T76820 | 7936988 | 7938013 | − | G | COG1638 | probable ABC transporter substrate-binding protein | | blr2170 |
| locusA | BJ6T76830 | 7938207 | 7939217 | − | G | COG0021 | putative transketolase beta subunit protein | | blr2169 |
| locusA | BJ6T76840 | 7939214 | 7940065 | − | G | COG0021 | putative transketolase alpha subunit protein | | blr2168 |
| locusA | BJ6T76850 | 7940090 | 7940854 | − | Q | COG1028 | oxidoreductase | | blr2167 |
| locusA | BJ6T76860 | 7940948 | 7941844 | + | K | COG0583 | transcriptional regulatory protein LysR family | | bll2166 |
| locusA | BJ6T76870 | 7942516 | 7942719 | | | | hypothetical protein | | |
| locusA | BJ6T76880 | 7943146 | 7943448 | | | | hypothetical protein | | |
| locusA | BJ6T76890 | 7944452 | 7945657 | + | L | COG3328 | putative transposase | | bll2163 |
| locusA | BJ6T76900 | 7945889 | 7946038 | + | S | COG2135 | hypothetical protein | | bll2162 |
| locusA | BJ6T76910 | 7946120 | 7946371 | | | | hypothetical protein | | |
| locusA | BJ6T76920 | 7946490 | 7947707 | − | I | COG1835 | hypothetical protein | | blr2157 |
| locusA | BJ6T76930 | 7947973 | 7948221 | − | | | hypothetical protein | | blr2156 |
| locusA | BJ6T76940 | 7948728 | 7949024 | | | | hypothetical protein | | |
| locusA | BJ6T76950 | 7949218 | 7949448 | + | | | hypothetical protein | | bll2154 |
| locusA | BJ6T76960 | 7949770 | 7950306 | − | | | hypothetical protein | | blr2153 |
| locusA | BJ6T76970 | 7950962 | 7951186 | − | L | COG2801 | hypothetical protein | | bsr2152 |
| locusA | BJ6T76980 | 7951219 | 7951428 | − | L | COG2801 | hypothetical protein | | bsr2151 |
| locusA | BJ6T76990 | 7951448 | 7952350 | − | | | hypothetical protein | | blr2150 |
| locusA | BJ6T77000 | 7952337 | 7953896 | − | | | hypothetical protein | | blr2149 |
| locusA | BJ6T77010 | 7954039 | 7955046 | − | H | COG0142 | farnesyl diphosphate synthase | *fpps* | blr2148 |
| locusA | BJ6T77020 | 7955192 | 7956535 | − | Q | COG2124 | cytochrome P-450 BJ-4 | *CYP117* | blr2147 |
| locusA | BJ6T77030 | 7956535 | 7957380 | − | Q | COG1028 | dehydrogenase | | blr2146 |
| locusA | BJ6T77040 | 7957385 | 7957675 | | Q | COG1028 | hypothetical protein | | |
| locusA | BJ6T77050 | 7957677 | 7958966 | − | Q | COG2124 | cytochrome P-450 BJ-3 | *CYP114* | blr2145 |
| locusA | BJ6T77060 | 7959061 | 7960266 | − | Q | COG2124 | cytochrome P-450 BJ-1 | *CYP112* | blr2144 |
| locusA | BJ6T77070 | 7960283 | 7960588 | − | Q | COG2124 | similar to cytochrome P450-family protein | | blr2143 |
| locusA | BJ6T77080 | 7960811 | 7961248 | + | | | hypothetical protein | | bll2142 |
| locusA | BJ6T77090 | 7961360 | 7961896 | + | | | hypothetical protein | | bll2141 |
| locusA | BJ6T77100 | 7961839 | 7962654 | − | | | hypothetical protein | | blr2140 |
| locusA | BJ6T77110 | 7963053 | 7963466 | + | L | COG2963 | putative transposase | | bll2139 |
| locusA | BJ6T77120 | 7963677 | 7964897 | + | L | COG0582 | probable site-specific integrase/recombinase | | bll2138 |
| locusA | BJ6T77130 | 7964897 | 7965517 | + | L | COG0582 | putative integrase/recombinase protein | | bll2137 |
| locusA | BJ6T77140 | 7966446 | 7967579 | − | K | COG1167 | putative aminotransferase | | blr2136 |
| locusA | BJ6T77150 | 7967599 | 7969176 | − | J | COG0143 | hypothetical protein | | blr2135 |
| locusA | BJ6T77160 | 7969178 | 7969507 | − | | | hypothetical protein | | blr2134 |
| locusA | BJ6T77170 | 7969532 | 7970311 | − | | | hypothetical protein | | blr2133 |
| locusA | BJ6T77180 | 7970277 | 7971581 | − | | | hypothetical protein | | blr2132 |
| locusA | BJ6T77190 | 7971578 | 7973080 | − | Q | COG3486 | probable oxygenase | | blr2131 |
| locusA | BJ6T77200 | 7973249 | 7973452 | | | | hypothetical protein | | |
| locusA | BJ6T77210 | 7973516 | 7973836 | − | N | COG3505 | similar to conjugal transfer protein traG | | blr2130 |
| locusA | BJ6T77220 | 7973946 | 7974182 | − | N | COG3505 | hypothetical protein | | bsr2129 |
| locusA | BJ6T77230 | 7974622 | 7975125 | + | | | hypothetical protein | | bll2128 |
| locusA | BJ6T77240 | 7975361 | 7975645 | − | | | hypothetical protein | | bsr2127 |
| locusA | BJ6T77250 | 7975630 | 7976214 | + | | | hypothetical protein | | bll2126 |
| locusA | BJ6T77260 | 7976508 | 7977425 | + | Q | COG2175 | probable dioxygenase | | bll2125 |
| locusA | BJ6T77270 | 7978337 | 7979092 | − | | | hypothetical protein | | blr2124 |
| locusA | BJ6T77280 | 7979116 | 7979682 | − | O | COG0265 | hypothetical protein | | blr2123 |
| locusA | BJ6T77290 | 7979840 | 7980010 | | | | hypothetical protein | | |
| locusA | BJ6T77300 | 7980385 | 7980669 | − | | | hypothetical protein | | blr2122 |
| locusA | BJ6T77310 | 7981383 | 7981790 | + | | | hypothetical protein | | bll2121 |
| locusA | BJ6T77320 | 7981926 | 7982132 | + | K | COG1278 | cold shock protein | *cspA* | bsl2120 |
| locusA | BJ6T77330 | 7982427 | 7982957 | − | P | COG3703 | probable cation transport protein | | blr2119 |
| locusA | BJ6T77340 | 7983003 | 7983572 | − | | | hypothetical protein | | blr2118 |
| locusA | BJ6T77350 | 7983686 | 7985026 | | | | hypothetical protein | | |
| locusA | BJ6T77360 | 7985058 | 7986974 | | | | hypothetical protein | | |
| locusA | BJ6T77370 | 7986981 | 7987529 | | | | hypothetical protein | | |
| locusA | BJ6T77380 | 7987673 | 7988797 | − | | | hypothetical protein | | blr2115 |
| locusA | BJ6T77390 | 7988810 | 7989130 | − | | | hypothetical protein | | blr2114 |
| locusA | BJ6T77400 | 7989127 | 7989501 | − | | | hypothetical protein | | blr2113 |
| locusA | BJ6T77410 | 7989926 | 7990156 | + | | | hypothetical protein | | bsl2112 |
| locusA | BJ6T77420 | 7990297 | 7990569 | − | | | hypothetical protein | | bsr2110 |
| locusA | BJ6T77430 | 7991408 | 7992091 | + | T | COG0664 | transcriptional regulatory protein Crp family | | bll2109 |
| locusA | BJ6T77440 | 7993713 | 8002292 | − | Q | COG1020 | probable peptide synthetase | | blr2108 |
| locusA | BJ6T77450 | 8002271 | 8002942 | − | Q | COG1020 | probable peptide synthetase | | blr2108 |
| locusA | BJ6T77460 | 8003516 | 8004064 | + | | | hypothetical protein | | bll2107 |
| locusA | BJ6T77470 | 8004189 | 8004479 | − | | | L-ectoine synthase | *ectC* | blr2106 |
| locusA | BJ6T77480 | 8005309 | 8005587 | − | | | hypothetical protein | | bsr2105 |
| locusA | BJ6T77490 | 8005984 | 8006187 | | | | hypothetical protein | | |
| locusA | BJ6T77500 | 8006533 | 8006733 | − | | | hypothetical protein | | bll2104 |
| locusA | BJ6T77510 | 8007402 | 8007761 | − | | | hypothetical protein | | blr2103 |
| locusA | BJ6T77520 | 8007774 | 8008625 | − | H | COG0414 | pantoate--beta-alanine ligase | *panC* | blr2102 |
| locusA | BJ6T77530 | 8008629 | 8009525 | − | H | COG0413 | 3-methyl-2-oxobutanoate hydroxymethyltransferase | *panB* | blr2101 |
| locusA | BJ6T77540 | 8009542 | 8009859 | − | | | hypothetical protein | | blr2100 |
| locusA | BJ6T77550 | 8009875 | 8010084 | | | | hypothetical protein | | |
| locusA | BJ6T77560 | 8010753 | 8011946 | − | H | COG0161 | adenosylmethionine-8-amino-7-oxononanoate aminotransferase | *bioA* | blr2099 |
| locusA | BJ6T77570 | 8012015 | 8012650 | − | H | COG0132 | dethiobiotin synthase | *bioD* | blr2098 |
| locusA | BJ6T77580 | 8012647 | 8013801 | − | H | COG0156 | 8-amino-7-oxononanoate synthase | *bioF* | blr2097 |
| locusA | BJ6T77590 | 8013878 | 8014216 | − | H | COG0853 | aspartate 1-decarboxylase precursor | *panD* | blr2096 |
| locusA | BJ6T77600 | 8014281 | 8015276 | − | H | COG0502 | biotin synthetase | *bioB* | blr2095 |
| locusA | BJ6T77610 | 8015643 | 8016026 | + | K | COG1802 | transcriptional regulatory protein GntR family | | bll2094 |

| | | | | | | | | | |
|---|---|---|---|---|---|---|---|---|---|
| locusA | BJ6T77620 | 8016162 | 8016479 | + | | | hypothetical protein | | bll2093 |
| locusA | BJ6T77630 | 8016618 | 8018027 | + | E | COG0147 | para-aminobenzoate synthase component I | *pabB* | bll2092 |
| locusA | BJ6T77640 | 8018024 | 8018620 | + | E | COG0512 | para-aminobenzoate synthase glutamine amidotransferase component II | *pabA* | bll2091 |
| locusA | BJ6T77650 | 8019291 | 8019764 | − | | | hypothetical protein | | blr2089 |
| locusA | BJ6T77660 | 8019761 | 8020048 | − | | | hypothetical protein | | bsr2088 |
| locusA | BJ6T77670 | 8020064 | 8020273 | | | | hypothetical protein | | |
| locusA | BJ6T77680 | 8020458 | 8023187 | + | | | hypothetical protein | | bll2087 |
| locusA | BJ6T77690 | 8023858 | 8024025 | + | | | hypothetical protein | | bsl2086 |
| locusA | BJ6T77700 | 8024326 | 8025558 | + | | | hypothetical protein | | bll2085 |
| locusA | BJ6T77710 | 8025666 | 8026001 | | | | hypothetical protein | | |
| locusA | BJ6T77720 | 8026159 | 8027496 | − | E | COG0174 | putative glutamine synthetase | | blr2084 |
| locusA | BJ6T77730 | 8027496 | 8028782 | − | I | COG0439 | hypothetical protein | | blr2083 |
| locusA | BJ6T77740 | 8028779 | 8029681 | − | | | hypothetical protein | | blr2082 |
| locusA | BJ6T77750 | 8029812 | 8030483 | − | R | COG2071 | putative amidotransferase subunit | | blr2081 |
| locusA | BJ6T77760 | 8030624 | 8031682 | − | I | COG3239 | putative dihydrorhizobitoxine desaturase | *rtxC* | blr2080 |
| locusA | BJ6T77770 | 8031714 | 8034125 | − | E | COG2873 | rtxA homolog | | blr2077 |
| locusA | BJ6T77780 | 8034731 | 8035060 | + | | | hypothetical protein | | bll2075 |
| locusA | BJ6T77790 | 8035872 | 8036165 | − | | | NoeE homolog | | blr2074 |
| locusA | BJ6T77800 | 8036589 | 8036840 | − | | | hypothetical protein | | blr2073 |
| locusA | BJ6T77810 | 8037915 | 8038100 | | | | hypothetical protein | | |
| locusA | BJ6T77820 | 8038116 | 8038472 | | | | hypothetical protein | | |
| locusA | BJ6T77830 | 8039209 | 8039460 | − | | | hypothetical protein | | bsr2072 |
| locusA | BJ6T77840 | 8040258 | 8040623 | − | | | similar to inosamine-phosphate amidinotransferas | | blr2071 |
| locusA | BJ6T77850 | 8040633 | 8040854 | | | | hypothetical protein | | |
| locusA | BJ6T77860 | 8041484 | 8041672 | | | | hypothetical protein | | |
| locusA | BJ6T77870 | 8044069 | 8046414 | − | E | COG0620 | 5-methyltetrahydropteroyltriglutamate-homocysteine S-methyltransferase | *metE* | blr2068 |
| locusA | BJ6T77880 | 8046513 | 8046788 | | | | hypothetical protein | | |
| locusA | BJ6T77890 | 8047705 | 8048430 | + | | | nodulate formation efficiency C protein | *nfeC* | bll2067 |
| locusA | BJ6T77900 | 8049565 | 8050278 | + | L | COG1525 | hypothetical protein | | bll2066 |
| locusA | BJ6T77910 | 8050287 | 8051030 | + | P | COG0288 | carbonic anhydrase | *icfA* | bll2065 |
| locusA | BJ6T77920 | 8051397 | 8051675 | + | | | hypothetical protein | | bsl2064 |
| locusA | BJ6T77930 | 8052927 | 8054093 | + | I | COG1960 | phenolhydroxylase homolog | *nrgC* | bll2063 |
| locusA | BJ6T77940 | 8054916 | 8055563 | − | Q | COG0500 | nodulation protein | *noeI* | blr2062 |
| locusA | BJ6T77950 | 8056514 | 8056828 | + | O | COG0234 | GroES3 chaperonin | *groES3* | bll2060 |
| locusA | BJ6T77960 | 8056913 | 8058553 | + | O | COG0459 | GroEL3 chaperonin | *groEL3* | bll2059 |
| locusA | BJ6T77970 | 8059793 | 8060308 | − | | | hypothetical protein | | blr2058 |
| locusA | BJ6T77980 | 8061701 | 8061862 | | | | hypothetical protein | | |
| locusA | BJ6T77990 | 8062802 | 8063407 | − | L | COG3547 | hypothetical protein | | blr2055 |
| locusA | BJ6T78000 | 8063370 | 8063846 | − | L | COG3547 | hypothetical protein | | blr2055 |
| locusA | BJ6T78010 | 8063936 | 8064823 | − | L | COG2801 | putative transposase | | blr2054 |
| locusA | BJ6T78020 | 8064786 | 8065040 | − | L | COG2801 | putative transposase | | blr2053 |
| locusA | BJ6T78030 | 8065217 | 8066461 | − | L | COG3344 | probable maturase; reverse transcriptase | | blr2052 |
| locusA | BJ6T78040 | 8067970 | 8068203 | − | | | hypothetical protein | | bsr2051 |
| locusA | BJ6T78050 | 8068671 | 8068970 | + | | | hypothetical protein | | bsl2050 |
| locusA | BJ6T78060 | 8069400 | 8070413 | + | E | COG0547 | anthranilate phosphoribosyltransferase | *trpD* | bll2049 |
| locusA | BJ6T78070 | 8070653 | 8070838 | + | | | hypothetical protein | | bsl2048 |
| locusA | BJ6T78080 | 8071251 | 8071616 | + | | | hypothetical protein | | bll2047 |
| locusA | BJ6T78090 | 8071967 | 8072746 | + | | | hypothetical protein | | bll2046 |
| locusA | BJ6T78100 | 8072774 | 8074084 | + | | | hypothetical protein | | bll2045 |
| locusA | BJ6T78110 | 8074623 | 8075021 | | | | hypothetical protein | | |
| locusA | BJ6T78120 | 8075565 | 8076242 | − | | | hypothetical protein | | blr2042 |
| locusA | BJ6T78130 | 8076227 | 8077378 | − | S | COG3791 | hypothetical protein | | blr2041 |
| locusA | BJ6T78140 | 8077465 | 8077845 | + | | | hypothetical protein | | bll2040 |
| locusA | BJ6T78150 | 8078055 | 8078456 | − | | | hypothetical protein | | blr2039 |
| locusA | BJ6T78160 | 8078589 | 8079455 | − | C | COG2086 | electron transfer flavoprotein beta chain | *fixA* | blr2038 |
| locusA | BJ6T78170 | 8079973 | 8081721 | − | K | COG3604 | nif-specific regulatory protein | *nifA* | blr2037 |
| locusA | BJ6T78180 | 8081974 | 8082810 | − | Q | COG1028 | oxidoreductase | *fixR* | blr2036 |
| locusA | BJ6T78190 | 8083539 | 8084117 | − | | | NodZ protein | *nodZ* | blr2035 |
| locusA | BJ6T78200 | 8084817 | 8086439 | − | O | COG2192 | NolO protein | *nolO* | blr2034 |
| locusA | BJ6T78210 | 8086460 | 8086843 | − | O | COG2192 | NolN protein | *nolN* | blr2033 |
| locusA | BJ6T78220 | 8086893 | 8087066 | − | | | hypothetical protein | *nolM* | bsr2032 |
| locusA | BJ6T78230 | 8087099 | 8087887 | − | R | COG0842 | NodJ protein | *nodJ* | blr2031 |
| locusA | BJ6T78240 | 8087891 | 8088805 | − | Q | COG1131 | transporter of Nod factors | *nodI* | blr2030 |
| locusA | BJ6T78250 | 8088813 | 8090522 | − | O | COG2192 | 6-O-carbamoyl transferase | *nodU* | blr2029 |
| locusA | BJ6T78260 | 8090536 | 8091165 | − | Q | COG0500 | N-methyl transferase | *nodS* | blr2028 |
| locusA | BJ6T78270 | 8091092 | 8092549 | − | | | chitin synthase | *nodC* | blr2027 |
| locusA | BJ6T78280 | 8092564 | 8093223 | − | | | de N-acatylase | *nodB* | blr2026 |
| locusA | BJ6T78290 | 8093220 | 8093852 | − | | | acyl transferase | *nodA* | blr2025 |
| locusA | BJ6T78295 | 8093894 | 8094532 | − | | | NodY protein | *nodY* | blr2024 |
| locusA | BJ6T78300 | 8095043 | 8095492 | + | L | COG3293 | putative transposase | | |
| locusA | BJ6T78310 | 8095532 | 8096425 | + | K | COG0583 | transcriptional regulatory protein LysR family | *nodD1* | bll2023 |
| locusA | BJ6T78320 | 8096620 | 8096973 | − | | | hypothetical protein | | blr2022 |
| locusA | BJ6T78330 | 8097248 | 8097538 | | | | hypothetical protein | | |
| locusA | BJ6T78340 | 8098638 | 8098847 | + | | | hypothetical protein | | bsl2020 |
| locusA | BJ6T78350 | 8099039 | 8099683 | + | K | COG0789 | transcriptional regulatory protein MerR family | *nolA* | bll2019 |
| locusA | BJ6T78360 | 8100000 | 8100398 | + | E | COG0683 | hypothetical protein | | bll2018 |
| locusA | BJ6T78370 | 8100505 | 8101098 | + | E | COG0683 | similar to ABC transporter, amino acid binding protein | | bll2017 |
| locusA | BJ6T78380 | 8102577 | 8102939 | + | | | NolY | *nolY* | bll2016 |
| locusA | BJ6T78390 | 8103046 | 8103273 | + | | | hypothetical protein | *nolZ* | bsl2015 |
| locusA | BJ6T78400 | 8103903 | 8104148 | + | | | hypothetical protein | | bsl2014 |
| locusA | BJ6T78410 | 8104971 | 8105696 | + | | | hypothetical protein | | bll2012 |
| locusA | BJ6T78420 | 8106334 | 8106696 | − | | | hypothetical protein | | blr2011 |
| locusA | BJ6T78430 | 8106668 | 8107072 | − | | | hypothetical protein | | bsr2010 |
| locusA | BJ6T78440 | 8107428 | 8108144 | + | R | COG0730 | hypothetical protein | | bll2009 |
| locusA | BJ6T78450 | 8108858 | 8109070 | − | | | hypothetical protein | | bsr2008 |
| locusA | BJ6T78460 | 8109787 | 8111124 | + | H | COG0635 | coproporphyrinogen III dehydrogenase | *hemN1* | bll2007 |
| locusA | BJ6T78470 | 8111533 | 8112051 | − | | | hypothetical protein | | blr2006 |
| locusA | BJ6T78480 | 8112303 | 8113169 | − | | | hypothetical protein | | bsr2005 |
| locusA | BJ6T78490 | 8113266 | 8114936 | + | | | hypothetical protein | | bll2003 |
| locusA | BJ6T78500 | 8115068 | 8115448 | + | L | COG2801 | putative transposase | | bll2002 |
| locusA | BJ6T78510 | 8115456 | 8116091 | − | | | hypothetical protein | | blr2001 |
| locusA | BJ6T78520 | 8116405 | 8116683 | − | | | hypothetical protein | | bsr1999 |
| locusA | BJ6T78530 | 8117684 | 8118097 | | | | hypothetical protein | | |
| locusA | BJ6T78540 | 8118307 | 8119251 | − | | | hypothetical protein | | blr1998 |
| locusA | BJ6T78550 | 8119611 | 8119985 | | | | hypothetical protein | | |
| locusA | BJ6T78560 | 8121435 | 8122475 | − | | | probable pectinesterase | | blr1994 |
| locusA | BJ6T78570 | 8122694 | 8123992 | − | | | probable polygalacturonase | | blr1993 |
| locusA | BJ6T78580 | 8124943 | 8125233 | − | | | hypothetical protein | | blr1992 |
| locusA | BJ6T78590 | 8125748 | 8126917 | − | | | hypothetical protein | | blr1991 |
| locusA | BJ6T78600 | 8126920 | 8130354 | − | | | hypothetical protein | | blr1990 |
| locusA | BJ6T78610 | 8130351 | 8130989 | − | | | hypothetical protein | | blr1989 |
| locusA | BJ6T78620 | 8130979 | 8132508 | − | | | hypothetical protein | | blr1988 |
| locusA | BJ6T78630 | 8132849 | 8133796 | + | L | COG3335 | putative transposase | | |
| locusA | BJ6T78640 | 8134100 | 8134351 | + | K | COG1396 | probable transcriptional regulator | | bsl1986 |
| locusA | BJ6T78650 | 8134351 | 8135670 | + | R | COG3550 | HipA protein | *hipA* | bll1985 |
| locusA | BJ6T78660 | 8135667 | 8135933 | | | | hypothetical protein | | |
| locusA | BJ6T78670 | 8135922 | 8136284 | − | | | putative transposase | | blr1984 |
| locusA | BJ6T78680 | 8137130 | 8137495 | + | | | hypothetical protein | | bll1981 |
| locusA | BJ6T78690 | 8137586 | 8138377 | + | | | hypothetical protein | | bll1980 |

| | | | | | | | | | |
|---|---|---|---|---|---|---|---|---|---|
| locusA | BJ6T78700 | 8138371 | 8138679 | + | | | hypothetical protein | | bll1979 |
| locusA | BJ6T78710 | 8138726 | 8139118 | + | | | hypothetical protein | | bll1978 |
| locusA | BJ6T78720 | 8139819 | 8141798 | + | C | COG1032 | hypothetical protein | | bll1977 |
| locusA | BJ6T78730 | 8142112 | 8142339 | − | | | hypothetical protein | | bsr1976 |
| locusA | BJ6T78740 | 8142723 | 8144069 | + | | | putative transposase | | |
| locusA | BJ6T78750 | 8144446 | 8144601 | | L | COG2801 | hypothetical protein | | |
| locusA | BJ6T78760 | 8144682 | 8144981 | − | L | COG2963 | putative transposase | | bsr1972 |
| locusA | BJ6T78770 | 8145187 | 8147505 | − | E | COG1506 | putative peptidase | | blr1971 |
| locusA | BJ6T78780 | 8147875 | 8148207 | − | | | hypothetical protein | | blr1970 |
| locusA | BJ6T78790 | 8148462 | 8149082 | − | L | COG3316 | hypothetical protein | | blr1969 |
| locusA | BJ6T78800 | 8149026 | 8149268 | + | | | hypothetical protein | | bsl1967 |
| locusA | BJ6T78810 | 8149290 | 8149550 | − | | | hypothetical protein | | bsr1966 |
| locusA | BJ6T78820 | 8150695 | 8151432 | − | | | putative sugar hydrolase | | blr1964 |
| locusA | BJ6T78830 | 8151673 | 8152041 | + | | | hypothetical protein | | bll1963 |
| locusA | BJ6T78840 | 8152361 | 8153542 | − | L | COG3344 | hypothetical protein | | blr1961 |
| locusA | BJ6T78850 | 8154134 | 8154325 | | | | hypothetical protein | | |
| locusA | BJ6T78860 | 8154343 | 8156796 | − | M | COG1452 | hypothetical protein | | blr1955 |
| locusA | BJ6T78870 | 8157184 | 8157900 | − | | | hypothetical protein | | blr1954 |
| locusA | BJ6T78880 | 8157979 | 8158431 | − | L | COG3293 | putative transposase | | blr1953 |
| locusA | BJ6T78890 | 8158347 | 8158739 | − | L | COG3293 | putative transposase | | |
| locusA | BJ6T78900 | 8160014 | 8160961 | − | L | COG3335 | putative transposase | | |
| locusA | BJ6T78910 | 8162726 | 8162986 | − | | | hypothetical protein | | bsr1909 |
| locusA | BJ6T78920 | 8163705 | 8163932 | + | L | COG3316 | hypothetical protein | | bsl1908 |
| locusA | BJ6T78930 | 8164190 | 8164444 | − | | | hypothetical protein | | bsr1907 |
| locusA | BJ6T78940 | 8164443 | 8164895 | + | K | COG0454 | N−acetyltransferase NrgA homolog | | bll1906 |
| locusA | BJ6T78950 | 8165253 | 8165564 | − | | | hypothetical protein | | blr1905 |
| locusA | BJ6T78960 | 8165599 | 8165769 | | | | hypothetical protein | | |
| locusA | BJ6T78970 | 8165826 | 8167571 | − | | | hypothetical protein | | blr1904 |
| locusA | BJ6T78980 | 8167764 | 8167952 | | | | hypothetical protein | | |
| locusA | BJ6T78990 | 8168210 | 8168455 | − | L | COG3436 | hypothetical protein | | bsr1903 |
| locusA | BJ6T79000 | 8169011 | 8169460 | | C | COG2141 | hypothetical protein | | |
| locusA | BJ6T79010 | 8169676 | 8170746 | | R | COG1853 | hypothetical protein | | |
| locusA | BJ6T79020 | 8171491 | 8171754 | | | | hypothetical protein | | |
| locusA | BJ6T79030 | 8173374 | 8174516 | | E | COG0687 | hypothetical protein | | |
| locusA | BJ6T79040 | 8175049 | 8175777 | | G | COG0235 | hypothetical protein | | |
| locusA | BJ6T79050 | 8176434 | 8176637 | | R | COG2130 | hypothetical protein | | |
| locusA | BJ6T79060 | 8176747 | 8177529 | | | | hypothetical protein | | |
| locusA | BJ6T79070 | 8177810 | 8178529 | | | | hypothetical protein | | |
| locusA | BJ6T79080 | 8178882 | 8179040 | | | | hypothetical protein | | |
| locusA | BJ6T79090 | 8180217 | 8180396 | | | | hypothetical protein | | |
| locusA | BJ6T79100 | 8180611 | 8180904 | | | | hypothetical protein | | |
| locusA | BJ6T79110 | 8181557 | 8182522 | | E | COG0111 | hypothetical protein | | |
| locusA | BJ6T79120 | 8182781 | 8182909 | | | | hypothetical protein | | |
| locusA | BJ6T79130 | 8182976 | 8184376 | | H | COG0161 | hypothetical protein | | |
| locusA | BJ6T79140 | 8184388 | 8185980 | | C | COG1012 | hypothetical protein | | |
| locusA | BJ6T79150 | 8186055 | 8186531 | | K | COG1522 | hypothetical protein | | |
| locusA | BJ6T79160 | 8187230 | 8188306 | | E | COG3842 | hypothetical protein | | |
| locusA | BJ6T79170 | 8188368 | 8189084 | | E | COG1177 | hypothetical protein | | |
| locusA | BJ6T79180 | 8189193 | 8189819 | | L | COG1961 | hypothetical protein | | |
| locusA | BJ6T79190 | 8190042 | 8190347 | | L | COG3436 | hypothetical protein | | |
| locusA | BJ6T79200 | 8190331 | 8190735 | | | | hypothetical protein | | |
| locusA | BJ6T79210 | 8190949 | 8191218 | | | | hypothetical protein | | |
| locusA | BJ6T79220 | 8191326 | 8191655 | | | | hypothetical protein | | |
| locusA | BJ6T79230 | 8191615 | 8191800 | | | | hypothetical protein | | |
| locusA | BJ6T79240 | 8192089 | 8192355 | | | | hypothetical protein | | |
| locusA | BJ6T79250 | 8192960 | 8193691 | | E | COG0665 | hypothetical protein | | |
| locusA | BJ6T79260 | 8193688 | 8194407 | | G | COG0235 | hypothetical protein | | |
| locusA | BJ6T79270 | 8194409 | 8197078 | | S | COG1262 | hypothetical protein | | |
| locusA | BJ6T79280 | 8197098 | 8197712 | | | | hypothetical protein | | |
| locusA | BJ6T79290 | 8197719 | 8198675 | | C | COG1052 | hypothetical protein | | |
| locusA | BJ6T79300 | 8198692 | 8199444 | | | | hypothetical protein | | |
| locusA | BJ6T79310 | 8200868 | 8201293 | | | | hypothetical protein | | |
| locusA | BJ6T79320 | 8201867 | 8202961 | − | E | COG1509 | hypothetical protein | | |
| locusA | BJ6T79330 | 8203634 | 8204080 | | | | hypothetical protein | | |
| locusA | BJ6T79340 | 8205014 | 8206462 | − | | | hypothetical protein | | |
| locusA | BJ6T79350 | 8207081 | 8207599 | − | J | COG1186 | hypothetical protein | | |
| locusA | BJ6T79360 | 8207713 | 8209077 | − | R | COG0612 | hypothetical zinc protease | | |
| locusA | BJ6T79370 | 8209074 | 8210366 | − | R | COG0612 | hypothetical zinc protease | | |
| locusA | BJ6T79380 | 8211277 | 8212086 | − | L | COG1484 | putative transposase | | |
| locusA | BJ6T79390 | 8212083 | 8213555 | − | | | putative transposase | | |
| locusA | BJ6T79400 | 8214666 | 8216441 | − | Q | COG1132 | ABC transporter, HlyB/MsbA family | | |
| locusA | BJ6T79410 | 8217155 | 8218147 | | O | COG0526 | hypothetical protein | | |
| locusA | BJ6T79420 | 8219174 | 8219497 | | G | COG2513 | hypothetical protein | | |
| locusA | BJ6T79430 | 8219824 | 8220168 | − | | | hypothetical protein | | |
| locusA | BJ6T79440 | 8220726 | 8220896 | − | | | hypothetical protein | | bsr1885 |
| locusA | BJ6T79450 | 8221032 | 8221307 | + | | | hypothetical protein | | bsl1884 |
| locusA | BJ6T79460 | 8221992 | 8223488 | − | K | COG1508 | RNA polymerase sigma−54 subunit | *rpoN1* | blr1883 |
| locusA | BJ6T79470 | 8223911 | 8224345 | − | | | hypothetical protein | | blr1882 |
| locusA | BJ6T79480 | 8224606 | 8224935 | − | | | hypothetical protein | | blr1881 |
| locusA | BJ6T79490 | 8225740 | 8226213 | − | K | COG2771 | transcriptional regulatory protein LuxR family | | blr1880 |
| locusA | BJ6T79500 | 8227626 | 8228240 | − | O | COG2214 | hypothetical protein | | blr1879 |
| locusA | BJ6T79510 | 8229647 | 8230975 | + | | | hypothetical protein | | bll1877 |
| locusA | BJ6T79520 | 8230991 | 8231554 | − | | | hypothetical protein | | blr1876 |
| locusA | BJ6T79530 | 8231660 | 8232076 | + | R | COG2940 | hypothetical protein | | bll1875 |
| locusA | BJ6T79540 | 8232816 | 8233268 | − | L | COG3293 | putative transposase | | bsr1874 |
| locusA | BJ6T79550 | 8233184 | 8233576 | − | L | COG3293 | putative transposase | | blr1873 |
| locusA | BJ6T79560 | 8234136 | 8235767 | + | | | hypothetical protein | | bll1872 |
| locusA | BJ6T79570 | 8236722 | 8236910 | | | | hypothetical protein | | |
| locusA | BJ6T79580 | 8237924 | 8238499 | − | | | hypothetical protein | | blr1869 |
| locusA | BJ6T79590 | 8239594 | 8239905 | − | | | hypothetical protein | | blr1868 |
| locusA | BJ6T79600 | 8240112 | 8240684 | − | L | COG3436 | hypothetical protein | | blr1867 |
| locusA | BJ6T79610 | 8240405 | 8241091 | − | L | COG3436 | hypothetical protein | | blr1866 |
| locusA | BJ6T79620 | 8241111 | 8241485 | − | L | COG2963 | hypothetical protein | | blr1865 |
| locusA | BJ6T79630 | 8241812 | 8243158 | + | G | COG0477 | citrate−proton symporter | *citA* | bll1864 |
| locusA | BJ6T79640 | 8243855 | 8244418 | + | | | putative transposase | | bll1861 |
| locusA | BJ6T79650 | 8244418 | 8244618 | + | | | hypothetical protein | | bll1860 |
| locusA | BJ6T79660 | 8244770 | 8245276 | − | | | hypothetical protein | | blr1859 |
| locusA | BJ6T79670 | 8245423 | 8245806 | + | | | hypothetical protein | | bll1858 |
| locusA | BJ6T79680 | 8246040 | 8246312 | + | | | hypothetical protein | | bsl1857 |
| locusA | BJ6T79690 | 8246390 | 8246683 | + | L | COG3293 | putative transposase | | bsl1856 |
| locusA | BJ6T79700 | 8246826 | 8247836 | + | L | COG1425 | putative transposase | | bll1855 |
| locusA | BJ6T79710 | 8248167 | 8250083 | − | | | hypothetical protein | | blr1854 |
| locusA | BJ6T79720 | 8250837 | 8251817 | − | Q | COG2124 | cytochrome P450 family protein | | blr1853 |
| locusA | BJ6T79730 | 8252629 | 8253510 | − | H | COG0414 | similar to pantoate−−beta−alanine ligase | | blr1852 |
| locusA | BJ6T79740 | 8253695 | 8255176 | − | S | COG1357 | hypothetical protein | | blr1851 |
| locusA | BJ6T79750 | 8255154 | 8255474 | − | | | hypothetical protein | | blr1850 |
| locusA | BJ6T79760 | 8255904 | 8256056 | + | | | hypothetical protein | | bsl1849 |
| locusA | BJ6T79770 | 8256583 | 8257491 | + | | | hypothetical protein | | bll1848 |
| locusA | BJ6T79780 | 8257954 | 8258388 | − | | | VirK protein homolog | | blr1847 |

| | | | | | | | | | |
|---|---|---|---|---|---|---|---|---|---|
| locusA | BJ6T79790 | 8259074 | 8260054 | + | | | hypothetical protein | | bll1846 |
| locusA | BJ6T79800 | 8261255 | 8261431 | + | | | hypothetical protein | | bsl1845 |
| locusA | BJ6T79810 | 8261648 | 8262028 | + | | | hypothetical protein | | bll1844 |
| locusA | BJ6T79820 | 8262068 | 8262757 | + | T | COG0745 | two-component response regulator | *basR* | bll1843 |
| locusA | BJ6T79830 | 8262784 | 8264238 | + | N | COG1450 | RhcC2 protein | *rhcC2* | bll1842 |
| locusA | BJ6T79840 | 8264249 | 8264911 | + | | | hypothetical protein | | bll1841 |
| locusA | BJ6T79850 | 8265107 | 8267422 | + | | | hypothetical protein | | bll1840 |
| locusA | BJ6T79860 | 8267715 | 8269097 | − | L | COG3344 | probable reverse transcriptase/maturase family protein | | blr1837 |
| locusA | BJ6T79870 | 8270095 | 8270577 | + | | | hypothetical protein | | bll1836 |
| locusA | BJ6T79880 | 8270631 | 8271341 | − | L | COG1484 | putative transposase | | blr1835 |
| locusA | BJ6T79890 | 8271347 | 8272705 | − | | | putative transposase | | blr1834 |
| locusA | BJ6T79900 | 8272809 | 8273651 | − | | | hypothetical protein | | blr1833 |
| locusA | BJ6T79910 | 8273352 | 8274686 | + | | | putative transposase | | bll1832 |
| locusA | BJ6T79920 | 8274816 | 8275022 | − | | | hypothetical protein | | bsr1831 |
| locusA | BJ6T79930 | 8275857 | 8276258 | − | | | hypothetical protein | | blr1830 |
| locusA | BJ6T79940 | 8277262 | 8278209 | − | L | COG3335 | putative transposase | | blr1829 |
| locusA | BJ6T79950 | 8278475 | 8278648 | + | | | hypothetical protein | | bsl1828 |
| locusA | BJ6T79960 | 8279034 | 8280065 | − | L | COG3547 | putative transposase | | blr1827 |
| locusA | BJ6T79970 | 8280388 | 8280720 | + | L | COG3547 | putative transposase | | bll1826 |
| locusA | BJ6T79980 | 8281055 | 8281477 | | | | hypothetical protein | | |
| locusA | BJ6T79990 | 8281898 | 8282416 | + | L | COG3293 | hypothetical protein | | bll1824 |
| locusA | BJ6T80000 | 8282208 | 8282657 | + | L | COG3293 | putative transposase | | bll1823 |
| locusA | BJ6T80010 | 8282667 | 8283704 | − | N | COG1377 | RhcU protein | *rhcU* | blr1822 |
| locusA | BJ6T80020 | 8283701 | 8284522 | − | N | COG1684 | RhcT protein | *rhcT* | blr1821 |
| locusA | BJ6T80030 | 8284533 | 8284808 | − | N | COG1987 | RhcS protein | *rhcS* | bsr1820 |
| locusA | BJ6T80040 | 8284811 | 8285476 | − | N | COG1338 | RhcR protein | *rhcR* | blr1819 |
| locusA | BJ6T80050 | 8285469 | 8286590 | − | N | COG1886 | RhcQ protein | *rhcQ* | blr1818 |
| locusA | BJ6T80060 | 8286587 | 8287123 | − | | | hypothetical protein | | blr1817 |
| locusA | BJ6T80070 | 8287099 | 8288454 | − | N | COG1157 | RhcN protein | *rhcN* | blr1816 |
| locusA | BJ6T80080 | 8288451 | 8289071 | − | N | COG1317 | nodulation protein | *nolV* | blr1815 |
| locusA | BJ6T80090 | 8289068 | 8289706 | − | | | nodulation protein | *nolU* | blr1814 |
| locusA | BJ6T80100 | 8289718 | 8290581 | − | N | COG1766 | RhcJ protein | *rhcJ* | blr1813 |
| locusA | BJ6T80110 | 8290590 | 8290895 | − | | | nodulation protein | *nolB* | blr1812 |
| locusA | BJ6T80120 | 8291504 | 8291965 | + | N | COG1450 | RhcC1 protein | *rhcC1* | bll1811 |
| locusA | BJ6T80130 | 8292246 | 8292749 | + | | | hypothetical protein | | bll1810 |
| locusA | BJ6T80140 | 8292695 | 8292928 | − | | | hypothetical protein | | bsr1809 |
| locusA | BJ6T80150 | 8293115 | 8293318 | + | | | hypothetical protein | | bsl1808 |
| locusA | BJ6T80160 | 8293889 | 8294836 | − | L | COG3335 | putative transposase | | blr1807 |
| locusA | BJ6T80170 | 8295108 | 8296562 | − | | | hypothetical protein | | blr1806 |
| locusA | BJ6T80180 | 8296813 | 8297373 | + | | | hypothetical protein | | bll1805 |
| locusA | BJ6T80190 | 8297471 | 8297779 | + | | | hypothetical protein | | bll1804 |
| locusA | BJ6T80200 | 8297822 | 8298013 | | | | hypothetical protein | | |
| locusA | BJ6T80210 | 8298130 | 8299023 | + | | | hypothetical protein | | bll1803 |
| locusA | BJ6T80220 | 8299057 | 8299593 | + | | | hypothetical protein | | bll1802 |
| locusA | BJ6T80230 | 8299596 | 8300024 | + | | | hypothetical protein | | bll1801 |
| locusA | BJ6T80240 | 8300034 | 8302133 | + | N | COG1298 | RhcV protein | *rhcV* | bll1800 |
| locusA | BJ6T80250 | 8302164 | 8302754 | + | R | COG0457 | hypothetical protein | | bll1799 |
| locusA | BJ6T80260 | 8302879 | 8303301 | + | | | hypothetical protein | | bll1798 |
| locusA | BJ6T80270 | 8303322 | 8303744 | + | | | hypothetical protein | | bll1797 |
| locusA | BJ6T80280 | 8303719 | 8304171 | + | | | hypothetical protein | | bll1796 |
| locusA | BJ6T80290 | 8304859 | 8306205 | + | R | COG0612 | similar to zinc protease | | bll1795 |
| locusA | BJ6T80300 | 8306361 | 8306924 | + | R | COG0612 | similar to protease | | bll1793 |
| locusA | BJ6T80310 | 8306836 | 8307648 | + | R | COG0612 | similar to zinc protease | | bll1792 |
| locusA | BJ6T80320 | 8308184 | 8308933 | + | | | hypothetical protein | | bll1791 |
| locusA | BJ6T80330 | 8308988 | 8309707 | + | | | hypothetical protein | | bll1790 |
| locusA | BJ6T80340 | 8309866 | 8310315 | − | | | hypothetical protein | | blr1789 |
| locusA | BJ6T80350 | 8310254 | 8310592 | − | | | hypothetical protein | | blr1788 |
| locusA | BJ6T80360 | 8310782 | 8311171 | − | | | hypothetical protein | | blr1787 |
| locusA | BJ6T80370 | 8312276 | 8312578 | + | | | hypothetical protein | | bll1786 |
| locusA | BJ6T80380 | 8313053 | 8313442 | − | | | hypothetical protein | | blr1785 |
| locusA | BJ6T80390 | 8314624 | 8315733 | + | P | COG1118 | molybdenum transport ATP-binding protein modC homolog | | bll1780 |
| locusA | BJ6T80400 | 8315790 | 8317058 | + | L | COG3328 | putative transposase | | bll1779 |
| locusA | BJ6T80410 | 8317179 | 8318135 | + | | | hypothetical protein | | bll1778 |
| locusA | BJ6T80420 | 8318087 | 8318641 | + | O | COG0450 | alkyl hydroperoxide reductase | *ahpC* | bll1777 |
| locusA | BJ6T80430 | 8318648 | 8319196 | + | S | COG2128 | alkyl hydroperoxide reductase | *ahpD* | bll1776 |
| locusA | BJ6T80440 | 8319408 | 8319704 | − | C | COG2440 | probable ferredoxin | *fixX* | bsr1775 |
| locusA | BJ6T80450 | 8319743 | 8321050 | − | C | COG0644 | flavoprotein | *fixC* | blr1774 |
| locusA | BJ6T80460 | 8321062 | 8322171 | − | C | COG2025 | electron transfer flavoprotein alpha chain | *fixB* | blr1773 |
| locusA | BJ6T80470 | 8322777 | 8322986 | + | | | hypothetical protein | | bsl1772 |
| locusA | BJ6T80480 | 8323122 | 8323463 | − | | | nitrogenase stabilizing/protective protein | *nifW* | blr1771 |
| locusA | BJ6T80490 | 8324118 | 8324894 | − | | | molybdenum processing protein | *nifQ* | blr1770 |
| locusA | BJ6T80500 | 8325041 | 8325925 | − | P | COG1348 | dinitrogenase reductase protein | *nifH* | blr1769 |
| locusA | BJ6T80510 | 8326107 | 8326316 | − | | | hypothetical protein | | blr1768 |
| locusA | BJ6T80520 | 8326643 | 8326918 | + | | | hypothetical protein | | bll1767 |
| locusA | BJ6T80530 | 8327140 | 8327871 | + | M | COG3047 | outer membrane protein | | bll1766 |
| locusA | BJ6T80540 | 8328277 | 8328624 | − | | | ferredoxin | *fer2* | blr1765 |
| locusA | BJ6T80550 | 8328775 | 8329071 | − | | | hypothetical protein | | bsr1764 |
| locusA | BJ6T80560 | 8329344 | 8329688 | − | | | hypothetical protein | | blr1763 |
| locusA | BJ6T80570 | 8329722 | 8330081 | | | | hypothetical protein | | |
| locusA | BJ6T80580 | 8330020 | 8330337 | | | | hypothetical protein | | |
| locusA | BJ6T80590 | 8330334 | 8330648 | − | | | iron-sulfur cofactor synthesis protein | *nifZ* | blr1761 |
| locusA | BJ6T80600 | 8330899 | 8331189 | − | C | COG1145 | hypothetical protein | *frxA* | bsr1760 |
| locusA | BJ6T80610 | 8331134 | 8332717 | − | R | COG0535 | FeMo cofactor biosynthesis protein | *nifB* | blr1759 |
| locusA | BJ6T80620 | 8332721 | 8332942 | − | | | hypothetical protein | | bsr1758 |
| locusA | BJ6T80630 | 8333381 | 8333605 | − | | | nitrogen fixation protein | *fixU* | bsr1757 |
| locusA | BJ6T80640 | 8333602 | 8334783 | − | E | COG1104 | nitrogenase metalloclusters biosynthesis protein | *nifS* | blr1756 |
| locusA | BJ6T80650 | 8335107 | 8335427 | − | S | COG0316 | R. etli iscN homolog | | blr1755 |
| locusA | BJ6T80660 | 8335687 | 8336277 | + | | | hypothetical protein | | bll1754 |
| locusA | BJ6T80670 | 8336299 | 8336682 | − | P | COG2146 | hypothetical protein | | blr1753 |
| locusA | BJ6T80680 | 8337281 | 8338015 | − | | | host-inducible protein A homolog | | blr1752 |
| locusA | BJ6T80690 | 8339007 | 8339300 | − | C | COG1145 | ferredoxin | *fer3* | bsr1750 |
| locusA | BJ6T80700 | 8339311 | 8339472 | − | | | hypothetical protein | | bsr1749 |
| locusA | BJ6T80710 | 8339525 | 8339989 | − | | | hypothetical protein | | blr1748 |
| locusA | BJ6T80720 | 8339996 | 8340391 | − | | | iron-molibdenum cofactor processing protein | *nifX* | blr1747 |
| locusA | BJ6T80730 | 8340388 | 8341797 | − | C | COG2710 | nitrogenase molybdenum-cofactor synthesis protein | *nifN* | blr1746 |
| locusA | BJ6T80740 | 8341807 | 8343450 | − | C | COG2710 | nitrogenase molybdenum-cofactor synthesis protein | *nifE* | blr1745 |
| locusA | BJ6T80750 | 8343543 | 8345099 | − | C | COG2710 | nitrogenase molybdenum-iron protein beta chain | *nifK* | blr1744 |
| locusA | BJ6T80760 | 8345165 | 8346667 | − | C | COG2710 | nitrogenase molybdenum-iron protein alpha chain | *nifD* | blr1743 |
| locusA | BJ6T80770 | 8347266 | 8347571 | + | | | putative transposase | | bll1742 |
| locusA | BJ6T80780 | 8347568 | 8348203 | + | L | COG2801 | putative transposase | | bll1741 |
| locusA | BJ6T80790 | 8348209 | 8348373 | + | | | putative transposase | | |
| locusA | BJ6T80800 | 8348479 | 8349426 | − | L | COG3335 | putative transposase | | blr1740 |
| locusA | BJ6T80810 | 8349687 | 8349881 | − | C | COG1145 | hypothetical protein | | bsr1739 |
| locusA | BJ6T80820 | 8350419 | 8351531 | − | E | COG0686 | alanine dehydrogenase | *aldA* | blr1738 |
| locusA | BJ6T80830 | 8351645 | 8352961 | − | O | COG0309 | hydrogenase expression/formation protein | *hypE* | blr1737 |
| locusA | BJ6T80840 | 8352958 | 8354109 | − | O | COG0409 | hydrogenase expression/formation protein | *hypD* | blr1736 |
| locusA | BJ6T80850 | 8354097 | 8355842 | − | O | COG0068 | hydrogenase maturation protein | *hypF* | blr1735 |
| locusA | BJ6T80860 | 8356126 | 8356953 | − | L | COG2801 | putative transposase | | |
| locusA | BJ6T80870 | 8356950 | 8357255 | − | | | putative transposase | | |

| | | | | | | | | | |
|---|---|---|---|---|---|---|---|---|---|
| locusA | BJ6T80880 | 8357293 | 8357595 | − | O | COG0378 | hydrogenase nickel incorporation protein HypB homolog | | blr1732 |
| locusA | BJ6T80890 | 8357592 | 8357894 | − | O | COG0378 | hydrogenase nickel incorporation protein HypB homolog | *hypB* | blr1731 |
| locusA | BJ6T80900 | 8358104 | 8358436 | − | R | COG0375 | hydrogenase nickel incorporation protein | *hypA* | blr1730 |
| locusA | BJ6T80910 | 8358438 | 8358791 | − | | | HupK protein homolog | | bsr1729 |
| locusA | BJ6T80920 | 8358760 | 8359161 | − | | | HupK protein homolog | *hupK* | blr1728 |
| locusA | BJ6T80930 | 8359627 | 8360178 | − | | | HupH protein homolog | *hupH* | blr1727 |
| locusA | BJ6T80940 | 8360480 | 8360872 | − | | | hypothetical protein | | blr1726 |
| locusA | BJ6T80950 | 8361056 | 8361328 | − | O | COG0298 | HupF protein homolog | *hupF* | bsr1725 |
| locusA | BJ6T80960 | 8361349 | 8361687 | − | C | COG0680 | HupD protein homolog | | blr1724 |
| locusA | BJ6T80970 | 8361753 | 8361947 | − | C | COG0680 | hypothetical protein | *hupD* | bsr1723 |
| locusA | BJ6T80980 | 8361982 | 8362674 | − | C | COG1969 | HupC protein homolog | *hupC* | blr1722 |
| locusA | BJ6T80990 | 8362679 | 8364469 | − | C | COG0374 | uptake hydrogenase large subunit homolog | *hupL* | blr1721 |
| locusA | BJ6T81000 | 8364483 | 8365574 | − | C | COG1740 | uptake hydrogenase small subunit precursor homolog | *hupS* | blr1720 |
| locusA | BJ6T81010 | 8366210 | 8366821 | − | P | COG0555 | molybdenum transport system permease protein | *modB* | blr1719 |
| locusA | BJ6T81020 | 8367318 | 8367503 | | | | hypothetical protein | | |
| locusA | BJ6T81030 | 8367929 | 8369245 | + | C | COG1301 | C4-dicarboxylate transport protein | *dctA* | bll1718 |
| locusA | BJ6T81040 | 8369401 | 8369826 | | | | hypothetical protein | | |
| locusA | BJ6T81050 | 8369736 | 8370128 | − | L | COG3293 | putative transposase | | |
| locusA | BJ6T81060 | 8370204 | 8371151 | − | L | COG3335 | putative transposase | | |
| locusA | BJ6T81070 | 8372414 | 8374816 | + | T | COG0642 | two component regulator | *nodV* | bll1715 |
| locusA | BJ6T81080 | 8374903 | 8375496 | + | T | COG2197 | two component regulator | *nodW* | bll1714 |
| locusA | BJ6T81090 | 8375581 | 8375967 | + | T | COG2197 | two-component response regulator | | bsl1713 |
| locusA | BJ6T81100 | 8376765 | 8378033 | − | L | COG3328 | putative transposase | | |
| locusA | BJ6T81110 | 8378163 | 8379368 | + | | | putative transposase | | |
| locusA | BJ6T81120 | 8379374 | 8380105 | + | L | COG1484 | putative transposase | | |
| locusA | BJ6T81130 | 8380114 | 8380692 | − | | | hypothetical protein | | blr1709 |
| locusA | BJ6T81140 | 8381150 | 8384827 | − | | | hypothetical protein | | blr1705 |
| locusA | BJ6T81150 | 8385100 | 8387352 | − | | | hypothetical protein | | blr1704 |
| locusA | BJ6T81160 | 8387523 | 8387834 | − | L | COG3316 | putative transposase | | |
| locusA | BJ6T81170 | 8387937 | 8389568 | + | L | COG1961 | hypothetical protein | | bll1703 |
| locusA | BJ6T81180 | 8389616 | 8390563 | − | L | COG3335 | putative transposase | | |
| locusA | BJ6T81190 | 8390948 | 8391952 | + | N | COG3505 | conjugal transfer protein | *trbG* | bll1701 |
| locusA | BJ6T81200 | 8392166 | 8393713 | − | L | COG3436 | putative transposase | | blr1700 |
| locusA | BJ6T81210 | 8393767 | 8394123 | − | L | COG3436 | hypothetical protein | | |
| locusA | BJ6T81220 | 8394674 | 8395018 | + | | | hypothetical protein | | |
| locusA | BJ6T81230 | 8395543 | 8395764 | | | | hypothetical protein | | |
| locusA | BJ6T81240 | 8396334 | 8396591 | + | S | COG3831 | hypothetical protein | | |
| locusA | BJ6T81250 | 8397326 | 8398015 | − | | | hypothetical protein | | |
| locusA | BJ6T81260 | 8399671 | 8402466 | − | | | hypothetical protein | | |
| locusA | BJ6T81270 | 8402668 | 8403378 | − | L | COG1484 | putative transposase | | |
| locusA | BJ6T81280 | 8403384 | 8404742 | − | | | putative transposase | | |
| locusA | BJ6T81290 | 8404969 | 8405274 | − | | | putative transposase | | |
| locusA | BJ6T81300 | 8405575 | 8405916 | + | | | probable suppressor protein | | bll1688 |
| locusA | BJ6T81310 | 8405913 | 8406416 | + | | | hypothetical protein | | bll1687 |
| locusA | BJ6T81320 | 8406748 | 8406981 | | M | COG1898 | hypothetical protein | | |
| locusA | BJ6T81330 | 8407141 | 8408520 | − | H | COG0161 | putative aminotransferase protein | | blr1686 |
| locusA | BJ6T81340 | 8408846 | 8409034 | | | | hypothetical protein | | |
| locusA | BJ6T81350 | 8409299 | 8409754 | − | | | putative transposase | | bll1685 |
| locusA | BJ6T81360 | 8409970 | 8410284 | + | | | hypothetical protein | | bll1684 |
| locusA | BJ6T81370 | 8410281 | 8410571 | − | | | hypothetical protein | | bsr1683 |
| locusA | BJ6T81380 | 8410751 | 8410936 | − | N | COG2948 | probable conjugal transfer protein | | |
| locusA | BJ6T81390 | 8411044 | 8411496 | − | L | COG3293 | putative transposase | | |
| locusA | BJ6T81400 | 8411412 | 8411804 | − | L | COG3293 | putative transposase | | |
| locusA | BJ6T81410 | 8411970 | 8412632 | − | E | COG0765 | probable ABC transporter permease protein | | blr1680 |
| locusA | BJ6T81420 | 8412629 | 8413144 | − | E | COG0765 | similar to ABC transporter permease protein | | blr1679 |
| locusA | BJ6T81430 | 8413253 | 8413444 | + | | | hypothetical protein | | bsl1678 |
| locusA | BJ6T81440 | 8413441 | 8413638 | − | | | hypothetical protein | | bsr1677 |
| locusA | BJ6T81450 | 8413803 | 8415665 | − | | | hypothetical protein | | blr1676 |
| locusA | BJ6T81460 | 8415909 | 8416325 | + | L | COG3316 | putative transposase | | |
| locusA | BJ6T81470 | 8416612 | 8417076 | − | | | hypothetical protein | | |
| locusA | BJ6T81480 | 8417066 | 8417455 | − | | | hypothetical protein | | |
| locusA | BJ6T81490 | 8417635 | 8418042 | | | | hypothetical protein | | |
| locusA | BJ6T81500 | 8419090 | 8419596 | − | Q | COG1020 | probable peptide synthetase | | |
| locusA | BJ6T81510 | 8419767 | 8420174 | − | | | hypothetical protein | | |
| locusA | BJ6T81520 | 8420316 | 8421071 | + | L | COG3316 | putative transposase | | bll1675 |
| locusA | BJ6T81530 | 8421284 | 8421751 | + | | | hypothetical protein | | bll1674 |
| locusA | BJ6T81540 | 8421874 | 8421949 | + | | | trnIleCAT | | |
| locusA | BJ6T81550 | 8422180 | 8422944 | | L | COG0084 | hypothetical protein | | |
| locusA | BJ6T81560 | 8422941 | 8424215 | | | | hypothetical protein | | |
| locusA | BJ6T81570 | 8424212 | 8425093 | | | | hypothetical protein | | |
| locusA | BJ6T81580 | 8425074 | 8426972 | | | | hypothetical protein | | |
| locusA | BJ6T81590 | 8427163 | 8428128 | | | | hypothetical protein | | |
| locusA | BJ6T81600 | 8428167 | 8428925 | | | | hypothetical protein | | |
| locusA | BJ6T81610 | 8428962 | 8430167 | − | | | hypothetical protein | | blr1667 |
| locusA | BJ6T81620 | 8430164 | 8431738 | − | | | hypothetical protein | | blr1666 |
| locusA | BJ6T81630 | 8431622 | 8432518 | + | | | hypothetical protein | | |
| locusA | BJ6T81640 | 8433173 | 8433619 | + | | | putative transposase | | blr1661 |
| locusA | BJ6T81650 | 8433628 | 8434101 | + | | | hypothetical protein | | bll1660 |
| locusA | BJ6T81660 | 8434922 | 8435413 | | | | hypothetical protein | | |
| locusA | BJ6T81670 | 8435526 | 8436050 | − | L | COG3547 | putative transposase | | blr1657 |
| locusA | BJ6T81680 | 8436374 | 8437180 | − | | | putative glycosyl hydrolase | | blr1656 |
| locusA | BJ6T81690 | 8437469 | 8438128 | − | L | COG3316 | putative transposase | | blr1655 |
| locusA | BJ6T81700 | 8438618 | 8438917 | + | S | COG2135 | hypothetical protein | | bsl1654 |
| locusA | BJ6T81710 | 8438925 | 8439266 | − | | | hypothetical protein | | blr1653 |
| locusA | BJ6T81720 | 8439546 | 8439749 | + | | | hypothetical protein | | bsl1652 |
| locusA | BJ6T81730 | 8439850 | 8440011 | + | | | hypothetical protein | | bsl1651 |
| locusA | BJ6T81740 | 8440464 | 8442002 | − | | | hypothetical protein | | blr1649 |
| locusA | BJ6T81750 | 8442143 | 8442511 | + | | | hypothetical protein | | bll1648 |
| locusA | BJ6T81760 | 8442938 | 8444284 | + | | | putative transposase | | bll1647 |
| locusA | BJ6T81770 | 8444300 | 8444752 | − | L | COG3293 | putative transposase | | bsr1646 |
| locusA | BJ6T81780 | 8444668 | 8445060 | − | L | COG3293 | putative transposase | | blr1645 |
| locusA | BJ6T81790 | 8445294 | 8445491 | | | | hypothetical protein | | |
| locusA | BJ6T81800 | 8445494 | 8445685 | | | | hypothetical protein | | |
| locusA | BJ6T81810 | 8446022 | 8446798 | − | T | COG2200 | hypothetical protein | | blr1644 |
| locusA | BJ6T81820 | 8447103 | 8447909 | + | | | hypothetical protein | | bll1643 |
| locusA | BJ6T81830 | 8448059 | 8449417 | + | | | putative transposase | | bll1642 |
| locusA | BJ6T81840 | 8449423 | 8450133 | + | L | COG1484 | putative transposase | | bll1641 |
| locusA | BJ6T81850 | 8450875 | 8451363 | − | | | hypothetical protein | | blr1640 |
| locusA | BJ6T81860 | 8452077 | 8452265 | | | | hypothetical protein | | |
| locusA | BJ6T81870 | 8452744 | 8452986 | | | | hypothetical protein | | |
| locusA | BJ6T81880 | 8453021 | 8453233 | | | | hypothetical protein | | |
| locusA | BJ6T81890 | 8453825 | 8454130 | − | | | hypothetical protein | | blr1638 |
| locusA | BJ6T81900 | 8454182 | 8454481 | | N | COG3549 | hypothetical protein | | |
| locusA | BJ6T81910 | 8454932 | 8455252 | + | | | hypothetical protein | | bll1636 |
| locusA | BJ6T81920 | 8455270 | 8455680 | − | | | probable site-specific integrase/recombinase | | blr1635 |
| locusA | BJ6T81930 | 8456057 | 8456413 | + | | | hypothetical protein | | bll1634 |
| locusA | BJ6T81940 | 8457034 | 8457474 | − | K | COG1278 | NoeD protein | *noeD* | blr1633 |
| locusA | BJ6T81950 | 8457807 | 8459633 | − | M | COG0449 | putative glucosamine synthase | *nodM* | blr1632 |
| locusA | BJ6T81960 | 8460967 | 8462052 | + | M | COG1089 | GDP-mannose 4,6-dehydratase | *noeL* | bll1631 |

| | | | | | | | | | |
|---|---|---|---|---|---|---|---|---|---|
| locusA | BJ6T81970 | 8462024 | 8463031 | + | M | COG0451 | GDP-fucose synthetase | *nolK* | bll1630 |
| locusA | BJ6T81980 | 8463306 | 8466422 | − | Q | COG0841 | multidrug resistance protein | | blr1629 |
| locusA | BJ6T81990 | 8467293 | 8467499 | − | | | hypothetical protein | | blr1627 |
| locusA | BJ6T82000 | 8467836 | 8468114 | | | | hypothetical protein | | |
| locusA | BJ6T82010 | 8468111 | 8468560 | | | | hypothetical protein | | |
| locusA | BJ6T82020 | 8468789 | 8469358 | − | | | hypothetical protein | | blr1625 |
| locusA | BJ6T82030 | 8472204 | 8472527 | + | | | hypothetical protein | | bll1623 |
| locusA | BJ6T82040 | 8473428 | 8474630 | − | N | COG2948 | conjugal transfer protein | *trbI* | blr1620 |
| locusA | BJ6T82050 | 8474627 | 8475661 | − | N | COG3504 | conjugal transfer protein | *trbG* | blr1619 |
| locusA | BJ6T82060 | 8475658 | 8476341 | − | N | COG3701 | probable conjugal transfer protein | *trbF* | blr1618 |
| locusA | BJ6T82070 | 8476434 | 8477273 | − | N | COG3846 | conjugal transfer protein | *trbL* | blr1617 |
| locusA | BJ6T82080 | 8477381 | 8478721 | − | | | hypothetical protein | | blr1616 |
| locusA | BJ6T82090 | 8478869 | 8479603 | − | K | COG1475 | hypothetical protein | | blr1615 |
| locusA | BJ6T82100 | 8479582 | 8480265 | − | K | COG1475 | hypothetical protein | | blr1614 |
| locusA | BJ6T82110 | 8480160 | 8481020 | − | K | COG1475 | hypothetical protein | | blr1613 |
| locusA | BJ6T82120 | 8481126 | 8482088 | − | | | antirestriction protein | *ardC* | blr1612 |
| locusA | BJ6T82130 | 8483159 | 8484025 | − | T | COG0642 | hypothetical protein | | blr1611 |
| locusA | BJ6T82140 | 8484009 | 8484845 | − | | | hypothetical protein | | blr1610 |
| locusA | BJ6T82150 | 8485493 | 8485729 | − | | | hypothetical protein | | bsr1607 |
| locusA | BJ6T82160 | 8486568 | 8486750 | | | | hypothetical protein | | |
| locusA | BJ6T82170 | 8487039 | 8488766 | − | R | COG1123 | ABC transporter ATP-binding protein | | blr1604 |
| locusA | BJ6T82180 | 8488763 | 8489644 | − | E | COG1173 | ABC transporter permease protein | | blr1603 |
| locusA | BJ6T82190 | 8489641 | 8490603 | − | E | COG0601 | ABC transporter permease protein | | blr1602 |
| locusA | BJ6T82200 | 8490600 | 8492288 | − | E | COG0747 | ABC transporter substrate-binding protein | | blr1601 |
| locusA | BJ6T82210 | 8492321 | 8493688 | − | C | COG2141 | nitrilotriacetate monooxygenase | | blr1600 |
| locusA | BJ6T82220 | 8494336 | 8494411 | + | | | trnIleCAT | | |
| locusA | BJ6T82230 | 8494524 | 8495690 | | | | hypothetical protein | | |
| locusA | BJ6T82240 | 8495909 | 8496199 | | | | hypothetical protein | | |
| locusA | BJ6T82250 | 8496259 | 8496603 | | | | hypothetical protein | | |
| locusA | BJ6T82260 | 8496615 | 8496905 | | | | hypothetical protein | | |
| locusA | BJ6T82270 | 8496915 | 8499569 | | | | hypothetical protein | | |
| locusA | BJ6T82280 | 8499566 | 8500918 | | | | hypothetical protein | | |
| locusA | BJ6T82290 | 8501261 | 8501710 | | | | hypothetical protein | | |
| locusA | BJ6T82300 | 8502251 | 8503012 | | I | COG1502 | hypothetical protein | | |
| locusA | BJ6T82310 | 8503009 | 8504859 | | | | hypothetical protein | | |
| locusA | BJ6T82320 | 8504856 | 8508908 | | | | hypothetical protein | | |
| locusA | BJ6T82330 | 8510121 | 8514059 | | L | COG1002 | hypothetical protein | | |
| locusA | BJ6T82340 | 8514056 | 8517199 | | K | COG0553 | hypothetical protein | | |
| locusA | BJ6T82350 | 8517199 | 8518299 | | L | COG0270 | hypothetical protein | | |
| locusA | BJ6T82360 | 8519039 | 8519968 | | | | hypothetical protein | | |
| locusA | BJ6T82370 | 8520006 | 8520440 | | | | hypothetical protein | | |
| locusA | BJ6T82380 | 8520878 | 8521987 | | | | hypothetical protein | | |
| locusA | BJ6T82390 | 8522172 | 8523110 | | | | hypothetical protein | | |
| locusA | BJ6T82400 | 8523119 | 8524294 | | | | hypothetical protein | | |
| locusA | BJ6T82410 | 8524570 | 8527224 | | | | hypothetical protein | | |
| locusA | BJ6T82420 | 8527924 | 8531376 | | | | hypothetical protein | | |
| locusA | BJ6T82430 | 8531918 | 8532148 | | | | hypothetical protein | | |
| locusA | BJ6T82440 | 8532275 | 8532970 | | L | COG1961 | hypothetical protein | | |
| locusA | BJ6T82450 | 8532957 | 8533229 | | | | hypothetical protein | | |
| locusA | BJ6T82460 | 8533299 | 8533586 | | | | hypothetical protein | | |
| locusA | BJ6T82470 | 8533583 | 8533966 | | | | hypothetical protein | | |
| locusA | BJ6T82480 | 8533966 | 8534157 | | | | hypothetical protein | | |
| locusA | BJ6T82490 | 8535006 | 8535728 | | | | hypothetical protein | | |
| locusA | BJ6T82500 | 8536224 | 8536742 | | | | hypothetical protein | | |
| locusA | BJ6T82510 | 8536749 | 8537051 | | | | hypothetical protein | | |
| locusA | BJ6T82520 | 8537557 | 8537943 | | | | hypothetical protein | | |
| locusA | BJ6T82530 | 8538185 | 8539384 | | L | COG0582 | probable site-specific integrase/recombinase | | |
| locusA | BJ6T82540 | 8540407 | 8542119 | + | | | hypothetical protein | | bll1595 |
| locusA | BJ6T82550 | 8542116 | 8542685 | + | | | hypothetical protein | | bll1594 |
| locusA | BJ6T82560 | 8542686 | 8545118 | + | | | hypothetical protein | | bll1593 |
| locusA | BJ6T82570 | 8546063 | 8547157 | + | | | hypothetical protein | | bll1592 |
| locusA | BJ6T82580 | 8547230 | 8547829 | + | | | hypothetical protein | | bll1591 |
| locusA | BJ6T82590 | 8548118 | 8548561 | − | | | hypothetical protein | | bsr1590 |
| locusA | BJ6T82600 | 8548660 | 8548896 | + | K | COG1396 | hypothetical protein | | bsl1589 |
| locusA | BJ6T82610 | 8548883 | 8549854 | − | S | COG1273 | conserved hypothetical protein | | blr1588 |
| locusA | BJ6T82620 | 8550005 | 8550382 | + | | | hypothetical protein | | bll1587 |
| locusA | BJ6T82630 | 8550944 | 8551309 | − | | | putative excisionase | | bll1586 |
| locusA | BJ6T82650 | 8551520 | 8552374 | + | L | COG0582 | probable site-specific integrase/recombinase | | bll1584 |
| locusA | BJ6T82660 | 8552885 | 8554618 | + | R | COG0666 | hypothetical protein | | bll1583 |
| locusA | BJ6T82670 | 8554615 | 8555418 | + | | | hypothetical protein | | bll1582 |
| locusA | BJ6T82680 | 8555430 | 8556551 | + | | | hypothetical protein | | bll1581 |
| locusA | BJ6T82690 | 8556842 | 8557447 | + | | | hypothetical protein | | bll1580 |
| locusA | BJ6T82700 | 8557613 | 8557960 | − | R | COG0637 | hypothetical protein | | blr1579 |
| locusA | BJ6T82710 | 8558001 | 8558390 | − | L | COG3436 | hypothetical protein | | blr1578 |
| locusA | BJ6T82720 | 8558532 | 8558738 | | | | hypothetical protein | | |
| locusA | BJ6T82730 | 8558914 | 8559078 | − | L | COG3436 | hypothetical protein | | bll1576 |
| locusA | BJ6T82740 | 8559106 | 8559492 | − | L | COG3436 | hypothetical protein | | blr1575 |
| locusA | BJ6T82750 | 8559719 | 8559970 | − | L | COG3436 | putative transposase | | bsr1574 |
| locusA | BJ6T82760 | 8559967 | 8560398 | − | | | hypothetical protein | | blr1573 |
| locusA | BJ6T82770 | 8560632 | 8562089 | + | | | hypothetical protein | | bll1572 |
| locusA | BJ6T82780 | 8562000 | 8563256 | − | | | hypothetical protein | | blr1571 |
| locusA | BJ6T82790 | 8563268 | 8563837 | − | | | hypothetical protein | | blr1571 |
| locusA | BJ6T82800 | 8563986 | 8576291 | + | | | hypothetical protein | | bll1570 |
| locusA | BJ6T82810 | 8576294 | 8584426 | + | | | hypothetical protein | | bll1569 |
| locusA | BJ6T82820 | 8584545 | 8585156 | + | | | hypothetical protein | | bll1568 |
| locusA | BJ6T82830 | 8585611 | 8586063 | + | | | hypothetical protein | | bll1567 |
| locusA | BJ6T82840 | 8586282 | 8587391 | + | | | hypothetical protein | | bll1566 |
| locusA | BJ6T82850 | 8587602 | 8590430 | − | | | hypothetical protein | | blr1564 |
| locusA | BJ6T82860 | 8590440 | 8590754 | − | | | hypothetical protein | | blr1563 |
| locusA | BJ6T82870 | 8591594 | 8591782 | − | | | hypothetical protein | | bsr1562 |
| locusA | BJ6T82880 | 8591769 | 8591993 | − | | | hypothetical protein | | bsr1561 |
| locusA | BJ6T82890 | 8592468 | 8592662 | + | | | hypothetical protein | | bll1560 |
| locusA | BJ6T82900 | 8593273 | 8593572 | | | | hypothetical protein | | |
| locusA | BJ6T82910 | 8593676 | 8594068 | + | | | hypothetical protein | | bll1558 |
| locusA | BJ6T82920 | 8594412 | 8594903 | + | | | hypothetical protein | | bll1557 |
| locusA | BJ6T82930 | 8595110 | 8595946 | − | T | COG0664 | hypothetical protein | | blr1556 |
| locusA | BJ6T82940 | 8596611 | 8596970 | + | C | COG3794 | hypothetical protein | | bll1555 |
| locusA | BJ6T82950 | 8596975 | 8597967 | − | | | hypothetical protein | | blr1554 |
| locusA | BJ6T82960 | 8598475 | 8599497 | − | P | COG1858 | methylamine utilization protein precursor | *mauG* | blr1552 |
| locusA | BJ6T82970 | 8599886 | 8600785 | − | T | COG2200 | hypothetical protein | | blr1551 |
| locusA | BJ6T82980 | 8600936 | 8601160 | | | | hypothetical protein | | |
| locusA | BJ6T82990 | 8601281 | 8601553 | | | | hypothetical protein | | |
| locusA | BJ6T83000 | 8601607 | 8602593 | − | N | COG0840 | hypothetical protein | | blr1551 |
| locusA | BJ6T83010 | 8603386 | 8604159 | | L | COG3293 | hypothetical protein | | |
| locusA | BJ6T83020 | 8605821 | 8606144 | | | | hypothetical protein | | |
| locusA | BJ6T83030 | 8606828 | 8607268 | | | | hypothetical protein | | |
| locusA | BJ6T83040 | 8607675 | 8607908 | − | | | hypothetical protein | | bsr1547 |
| locusA | BJ6T83050 | 8608384 | 8608587 | | | | hypothetical protein | | |
| locusA | BJ6T83060 | 8608723 | 8609121 | − | | | hypothetical protein | | blr1546 |

| | | | | | | | | | |
|---|---|---|---|---|---|---|---|---|---|
| locusA | BJ6T83070 | 8609003 | 8609395 | + | L | COG3293 | putative transposase | | |
| locusA | BJ6T83080 | 8609491 | 8609763 | + | L | COG3293 | putative transposase | | |
| locusA | BJ6T83090 | 8610188 | 8610601 | + | | | hypothetical protein | | bll1544 |
| locusA | BJ6T83100 | 8610615 | 8610929 | | | | hypothetical protein | | |
| locusA | BJ6T83110 | 8611885 | 8612154 | + | | | hypothetical protein | | bll1541 |
| locusA | BJ6T83120 | 8612359 | 8614416 | + | R | COG0824 | hypothetical protein | | bll1540 |
| | | | | | | | | | |
| locusC | BJ6T87770 | 9113996 | 9115237 | + | L | COG0582 | probable site-specific integrase/recombinase | | blr8174 |
| locusC | BJ6T87780 | 9115234 | 9116577 | + | | | hypothetical protein | | blr8175 |
| locusC | BJ6T87790 | 9116579 | 9117832 | + | | | hypothetical protein | | blr8176 |
| locusC | BJ6T87800 | 9117923 | 9120412 | − | | | hypothetical protein | | bll8177 |
| locusC | BJ6T87810 | 9120409 | 9120633 | | | | hypothetical protein | | |
| locusC | BJ6T87820 | 9120723 | 9121499 | − | | | hypothetical protein | | bll8178 |
| locusC | BJ6T87830 | 9121686 | 9122240 | | | | hypothetical protein | | |
| locusC | BJ6T87840 | 9122359 | 9124146 | + | | | hypothetical protein | | blr8180 |
| locusC | BJ6T87850 | 9124187 | 9125296 | + | L | COG3335 | putative transposase | | blr8181 |
| locusC | BJ6T87860 | 9125307 | 9126071 | − | L | COG3293 | putative transposase | | bll8182 |
| locusC | BJ6T87870 | 9126630 | 9127694 | − | L | COG3547 | putative transposase | | bll8183 |
| locusC | BJ6T87880 | 9127952 | 9128536 | − | | | hypothetical protein | | bll8184 |
| locusC | BJ6T87890 | 9128783 | 9129151 | + | K | COG1309 | transcriptional regulatory protein TetR family | | blr8185 |
| locusC | BJ6T87900 | 9129127 | 9129408 | + | | | transcriptional regulatory protein TetR family | | blr8185 |
| locusC | BJ6T87910 | 9129568 | 9130215 | + | S | COG1376 | hypothetical protein | | blr8186 |
| locusC | BJ6T87920 | 9130320 | 9131198 | + | Q | COG1028 | dehydrogenase | | blr8187 |
| locusC | BJ6T87930 | 9131195 | 9132043 | + | R | COG0596 | putative dehalogenase | | blr8188 |
| locusC | BJ6T87940 | 9132056 | 9132679 | + | E | COG0346 | hypothetical protein | | blr8189 |
| locusC | BJ6T87950 | 9132952 | 9133251 | + | | | hypothetical protein | | blr8190 |
| locusC | BJ6T87960 | 9133715 | 9134068 | | | | hypothetical protein | | |
| locusC | BJ6T87970 | 9134131 | 9135036 | − | | | putative transposase | | bll8192 |
| locusC | BJ6T87980 | 9135702 | 9135986 | − | K | COG1396 | probable transcriptional regulator | | |
| locusC | BJ6T87990 | 9136311 | 9136640 | | | | hypothetical protein | | |
| locusC | BJ6T88000 | 9136660 | 9137544 | | | | hypothetical protein | | |
| locusC | BJ6T88010 | 9138011 | 9138676 | | | | hypothetical protein | | |
| locusC | BJ6T88020 | 9138673 | 9138990 | | | | hypothetical protein | | |
| locusC | BJ6T88030 | 9138980 | 9139720 | | | | hypothetical protein | | |
| locusC | BJ6T88040 | 9139764 | 9140726 | | | | hypothetical protein | | |
| locusC | BJ6T88050 | 9141059 | 9141235 | | | | hypothetical protein | | |
| locusC | BJ6T88060 | 9141814 | 9142680 | + | L | COG0582 | probable site-specific integrase/recombinase | | blr8193 |
| locusC | BJ6T88070 | 9142373 | 9143098 | + | L | COG3436 | putative transposase | | blr8194 |
| locusC | BJ6T88080 | 9143335 | 9144663 | − | L | COG3344 | probable group II intron, maturase/reverse transcriptase | | bll8195 |
| locusC | BJ6T88090 | 9144599 | 9145435 | − | | | putative outer-membrane immunogenic protein precursor | | bll8196 |
| locusC | BJ6T88100 | 9145879 | 9146547 | + | D | COG1192 | probable partition protein | | blr8197 |
| locusC | BJ6T88110 | 9146735 | 9147547 | + | | | hypothetical protein | | blr8198 |
| locusC | BJ6T88120 | 9147552 | 9148415 | − | L | COG1484 | putative transposase | | bll8199 |
| locusC | BJ6T88130 | 9148415 | 9148969 | − | | | putative transposase | | bll8200 |
| locusC | BJ6T88140 | 9149185 | 9150696 | − | | | putative transposase | | |
| locusC | BJ6T88150 | 9151885 | 9152712 | − | L | COG2801 | putative transposase | | blr8261 |
| locusC | BJ6T88160 | 9152709 | 9153014 | − | | | putative transposase | | blr8260 |
| locusC | BJ6T88170 | 9153178 | 9153558 | + | | | hypothetical protein | | bll8259 |
| locusC | BJ6T88180 | 9153515 | 9153907 | + | | | hypothetical protein | | bll8258 |
| locusC | BJ6T88190 | 9153922 | 9155349 | + | R | COG3378 | hypothetical protein | | bll8257 |
| locusC | BJ6T88200 | 9155750 | 9156301 | + | | | hypothetical protein | | bll8256 |
| locusC | BJ6T88210 | 9156499 | 9157032 | + | | | hypothetical protein | | bll8255 |
| locusC | BJ6T88220 | 9157032 | 9157676 | | | | hypothetical protein | | |
| locusC | BJ6T88230 | 9157785 | 9158063 | + | R | COG3740 | hypothetical protein | | bsl8253 |
| locusC | BJ6T88240 | 9158066 | 9158275 | + | | | hypothetical protein | | bsl8252 |
| locusC | BJ6T88250 | 9158375 | 9158740 | + | | | hypothetical protein | | bll8251 |
| locusC | BJ6T88260 | 9158877 | 9160307 | + | | | hypothetical protein | | bll8250 |
| locusC | BJ6T88270 | 9160324 | 9160527 | + | K | COG1278 | cold shock protein | *cspA* | bsl8249 |
| locusC | BJ6T88280 | 9160913 | 9161143 | | | | hypothetical protein | | |
| locusC | BJ6T88290 | 9162447 | 9162764 | + | | | hypothetical protein | | bll8248 |
| locusC | BJ6T88300 | 9162790 | 9163074 | − | | | hypothetical protein | | bsr8247 |
| locusC | BJ6T88310 | 9163382 | 9163660 | | | | hypothetical protein | | |
| locusC | BJ6T88320 | 9163979 | 9164221 | | | | hypothetical protein | | |
| locusC | BJ6T88330 | 9164225 | 9164461 | | | | hypothetical protein | | |
| locusC | BJ6T88340 | 9164461 | 9164703 | − | | | hypothetical protein | | bsr8246 |
| locusC | BJ6T88350 | 9165119 | 9165376 | | | | hypothetical protein | | |
| locusC | BJ6T88360 | 9166024 | 9170349 | + | | | hypothetical protein | | bll8244 |
| locusC | BJ6T88370 | 9171084 | 9171341 | − | S | COG3831 | hypothetical protein | | bsr8241 |
| locusC | BJ6T88380 | 9171940 | 9172389 | + | L | COG3293 | putative transposase | | |
| locusC | BJ6T88390 | 9172746 | 9172967 | | | | hypothetical protein | | |
| locusC | BJ6T88400 | 9173492 | 9173836 | − | | | hypothetical protein | | blr8240 |
| locusC | BJ6T88410 | 9174112 | 9174390 | + | L | COG2963 | hypothetical protein | | bll8239 |
| locusC | BJ6T88420 | 9174387 | 9174743 | + | L | COG3436 | hypothetical protein | | bll8238 |
| locusC | BJ6T88430 | 9174797 | 9175069 | + | | | hypothetical protein | | bll8237 |
| locusC | BJ6T88440 | 9175148 | 9175975 | − | L | COG2801 | putative transposase | | |
| locusC | BJ6T88450 | 9175972 | 9176277 | − | | | putative transposase | | |
| locusC | BJ6T88460 | 9176477 | 9176854 | − | L | COG1425 | hypothetical protein | | blr8234 |
| locusC | BJ6T88470 | 9177303 | 9177560 | − | L | COG2963 | putative transposase | | blr8233 |
| locusC | BJ6T88480 | 9177579 | 9177746 | − | | | putative transposase | | blr8233 |
| locusC | BJ6T88490 | 9179008 | 9180114 | + | E | COG1509 | hypothetical protein | | bll8232 |
| locusC | BJ6T88500 | 9180122 | 9181744 | + | E | COG0405 | putative gamma-glutamyltranspeptidase | | bll8231 |
| locusC | BJ6T88510 | 9181741 | 9182604 | + | | | hypothetical protein | | bll8230 |
| locusC | BJ6T88520 | 9182595 | 9183641 | + | M | COG1181 | hypothetical protein | | bll8229 |
| locusC | BJ6T88530 | 9183853 | 9184032 | + | | | hypothetical protein | | bsl8228 |
| locusC | BJ6T88540 | 9184193 | 9184468 | + | | | hypothetical protein | | bll8227 |
| locusC | BJ6T88550 | 9184665 | 9184931 | | | | hypothetical protein | | |
| locusC | BJ6T88560 | 9185123 | 9185452 | − | | | hypothetical protein | | blr8224 |
| locusC | BJ6T88570 | 9185788 | 9186111 | + | | | hypothetical protein | | bll8223 |
| locusC | BJ6T88580 | 9186223 | 9186624 | − | L | COG1425 | putative transposase | | blr8222 |
| locusC | BJ6T88590 | 9186802 | 9187029 | − | L | COG2801 | putative transposase | | bsr8221 |
| locusC | BJ6T88600 | 9187152 | 9187913 | − | L | COG1484 | putative transposase | | blr8220 |
| locusC | BJ6T88610 | 9187932 | 9189473 | − | | | putative transposase | | blr8219 |
| locusC | BJ6T88620 | 9189579 | 9189881 | − | L | COG2801 | putative transposase | | blr8218 |
| locusC | BJ6T88630 | 9189602 | 9190228 | − | L | COG2801 | hypothetical protein | | blr8217 |
| locusC | BJ6T88640 | 9190225 | 9190530 | − | | | putative transposase | | blr8216 |
| locusC | BJ6T88650 | 9190585 | 9190950 | − | L | COG1425 | putative transposase | | blr8215 |
| locusC | BJ6T88660 | 9190996 | 9191256 | − | L | COG2963 | putative transposase | | bsr8214 |
| locusC | BJ6T88670 | 9191469 | 9191681 | + | C | COG1012 | hypothetical protein | | bsr8213 |
| locusC | BJ6T88680 | 9191927 | 9193060 | + | H | COG0654 | putative monooxygenase | | bll8212 |
| locusC | BJ6T88690 | 9193406 | 9193630 | + | L | COG3316 | hypothetical protein | | bsl8210 |
| locusC | BJ6T88700 | 9193492 | 9194109 | + | L | COG3316 | hypothetical protein | | bll8209 |
| locusC | BJ6T88710 | 9194033 | 9194227 | + | I | COG2084 | hypothetical protein | | bsl8208 |
| locusC | BJ6T88720 | 9194197 | 9194526 | + | | | hypothetical protein | | bll8207 |
| locusC | BJ6T88730 | 9194533 | 9195093 | + | L | COG3344 | probable maturase; reverse transcriptase | | bll8206 |
| locusC | BJ6T88740 | 9195020 | 9195292 | + | L | COG3344 | probable reverse transcriptase/maturase family protein | | |
| locusC | BJ6T88750 | 9195424 | 9196011 | + | | | probable maturase; reverse transcriptase | | bll8205 |
| locusC | BJ6T88760 | 9196110 | 9196565 | − | | | hypothetical protein | | blr8204 |
| locusC | BJ6T88770 | 9196738 | 9197142 | + | | | hypothetical protein | | bll8203 |
| locusC | BJ6T88780 | 9197378 | 9197644 | + | | | hypothetical protein | | bll8202 |

| | | | | | | | | | |
|---|---|---|---|---|---|---|---|---|---|
| locusC | BJ6T88790 | 9198617 | 9199156 | + | | | hypothetical protein | | bll8201 |
| locusC | BJ6T88800 | 9199454 | 9200458 | + | | | putative transposase | | bll8200 |
| locusC | BJ6T88810 | 9200528 | 9201760 | − | | | putative transposase | | |
| locusC | BJ6T88820 | 9202391 | 9202693 | | | | hypothetical protein | | |
| locusC | BJ6T88830 | 9203199 | 9203465 | | | | hypothetical protein | | |
| locusC | BJ6T88840 | 9203923 | 9204240 | | | | hypothetical protein | | |
| locusC | BJ6T88850 | 9204343 | 9204639 | − | | | hypothetical protein | | bsl8315 |
| locusC | BJ6T88860 | 9204953 | 9205195 | + | | | hypothetical protein | | |
| locusC | BJ6T88870 | 9205374 | 9205688 | + | L | COG2801 | hypothetical protein | | bsr8316 |
| locusC | BJ6T88880 | 9206723 | 9206893 | | | | hypothetical protein | | |
| locusC | BJ6T00010 | 123 | 428 | + | | | hypothetical protein | | blr0001 |
| locusC | BJ6T00020 | 484 | 1023 | + | | | hypothetical protein | | blr0002 |
| locusC | BJ6T00030 | 1544 | 1795 | | | | hypothetical protein | | |
| locusC | BJ6T00040 | 2945 | 3193 | + | | | hypothetical protein | | blr0003 |
| locusC | BJ6T00050 | 3160 | 3681 | + | | | hypothetical protein | | blr0003 |
| locusC | BJ6T00060 | 4425 | 4682 | − | | | hypothetical protein | | bll0005 |
| locusC | BJ6T00070 | 4970 | 6043 | − | F | COG0208 | ribonucleoside-diphosphate reductase beta subunit | *nrdB* | bll0006 |
| locusC | BJ6T00080 | 6034 | 7977 | − | F | COG0209 | probable ribonucleoside-diphosphate reductase | | bll0007 |
| locusC | BJ6T00090 | 8414 | 8668 | | | | hypothetical protein | | |
| locusC | BJ6T00100 | 8959 | 9465 | − | K | COG0454 | putative acetyltransferase | | bll0009 |
| locusC | BJ6T00110 | 10342 | 10740 | − | | | hypothetical protein | | bll0010 |
| locusC | BJ6T00120 | 10901 | 11086 | | | | hypothetical protein | | |
| locusC | BJ6T00130 | 11785 | 12156 | + | L | COG1961 | hypothetical protein | | blr0012 |
| locusC | BJ6T00140 | 12278 | 12811 | + | L | COG1961 | putative transposase | | blr0013 |
| locusC | BJ6T00150 | 12768 | 12923 | | | | hypothetical protein | | |
| locusC | BJ6T00160 | 13094 | 13417 | | | | hypothetical protein | | |
| locusC | BJ6T00170 | 13676 | 14065 | − | | | putative transposase | | bll0015 |
| locusC | BJ6T00180 | 14062 | 14373 | − | | | hypothetical protein | | bsl0016 |
| locusC | BJ6T00190 | 14735 | 15040 | + | | | putative transposase | | bll0017 |
| locusC | BJ6T00200 | 15037 | 15864 | + | L | COG2801 | putative transposase | | bll0018 |
| locusC | BJ6T00210 | 16185 | 16514 | + | | | hypothetical protein | | bll0019 |
| locusC | BJ6T00220 | 17135 | 18367 | − | Q | COG2124 | hypothetical protein | | bll0020 |
| locusC | BJ6T00230 | 18493 | 20091 | − | | | hypothetical protein | | bll0021 |
| locusC | BJ6T00240 | 21521 | 21811 | + | | | hypothetical protein | | bsr0022 |
| locusC | BJ6T00250 | 22133 | 23077 | + | L | COG3335 | putative transposase | | blr0023 |
| locusC | BJ6T00260 | 23738 | 23980 | − | L | COG1484 | hypothetical protein | | blr0024 |
| locusC | BJ6T00270 | 24722 | 26236 | − | | | 67 kDa Myosin-crossreactive streptococcal antigen homolog | | bll0025 |
| locusC | BJ6T00280 | 26254 | 26481 | − | | | 67 kDa Myosin-crossreactive streptococcal antigen homolog | | bll0025 |
| locusC | BJ6T00290 | 26527 | 27285 | − | Q | COG1028 | probable dehydrogenase | | bll0026 |
| locusC | BJ6T00300 | 27735 | 27953 | + | | | hypothetical protein | | bsr0027 |
| locusC | BJ6T00310 | 28522 | 29310 | + | | | hypothetical protein | | blr0028 |
| locusC | BJ6T00320 | 29407 | 29694 | + | | | hypothetical protein | | bsr0029 |
| locusC | BJ6T00330 | 29787 | 31367 | + | L | COG1961 | recombinase | | blr0030 |
| locusC | BJ6T00340 | 31432 | 31671 | + | | | hypothetical protein | | bll0031 |
| locusC | BJ6T00350 | 31754 | 31939 | − | | | hypothetical protein | | bsl0032 |
| locusC | BJ6T00360 | 32234 | 32467 | + | G | COG0726 | hypothetical protein | | bsr0033 |
| locusC | BJ6T00370 | 32880 | 33305 | + | L | COG2963 | putative transposase | | blr0034 |
| locusC | BJ6T00380 | 33302 | 33658 | + | L | COG3436 | hypothetical protein | | blr0035 |
| locusC | BJ6T00390 | 33645 | 33947 | + | E | COG0834 | hypothetical protein | | bsr0036 |
| locusC | BJ6T00400 | 33960 | 34316 | + | E | COG0765 | hypothetical protein | | blr0037 |
| locusC | BJ6T00410 | 34574 | 34855 | + | K | COG1396 | probable transcriptional regulator | | bsr0038 |
| locusC | BJ6T00420 | 35822 | 36217 | − | S | COG3791 | hypothetical protein | | bll0040 |
| locusC | BJ6T00430 | 36852 | 37238 | − | | | hypothetical protein | | bll0042 |
| locusC | BJ6T00440 | 37299 | 39281 | − | N | COG3505 | conjugal transfer protein | *traG* | bll0043 |
| locusC | BJ6T00450 | 39323 | 40498 | − | N | COG3843 | hypothetical protein | | bll0044 |
| locusC | BJ6T00460 | 41530 | 42072 | − | M | COG0741 | probable transglycosylase | | bll0045 |
| locusC | BJ6T00470 | 42442 | 42777 | − | | | hypothetical protein | | bll0046 |
| locusC | BJ6T00480 | 42812 | 43357 | − | N | COG0681 | conjugal transfer protein precursor | *traF* | bll0047 |
| locusC | BJ6T00490 | 43354 | 43872 | − | | | hypothetical protein | | bll0048 |
| locusC | BJ6T00500 | 43859 | 44125 | − | | | hypothetical protein | | bsl0049 |
| locusC | BJ6T00510 | 44122 | 44760 | − | D | COG1192 | putative partition protein | | bll0050 |
| locusC | BJ6T00520 | 44757 | 45872 | − | | | replication protein A | *repA* | bll0051 |
| locusC | BJ6T00530 | 45886 | 46137 | − | | | hypothetical protein | | bll0052 |
| locusC | BJ6T00540 | 46341 | 46856 | − | | | hypothetical protein | | bll0053 |
| locusC | BJ6T00550 | 46918 | 47127 | | | | hypothetical protein | | |
| locusC | BJ6T00560 | 47247 | 47510 | − | | | hypothetical protein | | bsl0054 |
| locusC | BJ6T00570 | 47760 | 47975 | + | K | COG1396 | probable transcriptional regulator | | bsr0055 |
| locusC | BJ6T00580 | 48139 | 48549 | + | | | hypothetical protein | | blr0056 |
| locusC | BJ6T00590 | 48666 | 48992 | − | | | hypothetical protein | | bll0057 |
| locusC | BJ6T00600 | 49041 | 49340 | + | | | hypothetical protein | | |
| locusC | BJ6T00610 | 49700 | 50041 | + | | | hypothetical protein | | blr0058 |
| locusC | BJ6T00620 | 50073 | 50279 | − | K | COG1278 | cold shock protein | *cspA* | bsl0059 |
| locusC | BJ6T00630 | 50714 | 51487 | + | Q | COG0500 | hypothetical protein | | blr0060 |
| locusC | BJ6T00640 | 51536 | 51958 | − | | | hypothetical protein | | bll0061 |
| locusC | BJ6T00650 | 52254 | 52922 | + | | | Atc1 protein | *atc1* | blr0062 |
| locusC | BJ6T00660 | 52998 | 53927 | − | | | hypothetical protein | | bll0063 |
| locusC | BJ6T00670 | 54254 | 55306 | − | | | hypothetical protein | | bll0064 |
| locusC | BJ6T00680 | 55369 | 57462 | − | K | COG1475 | putative DNA-binding protein | | bll0065 |
| locusC | BJ6T00690 | 57635 | 58813 | − | | | hypothetical protein | | bll0066 |
| locusC | BJ6T00700 | 59388 | 59606 | | | | hypothetical protein | | |
| locusC | BJ6T00710 | 59734 | 59949 | + | | | hypothetical protein | | bsr0067 |
| locusC | BJ6T00720 | 60216 | 61622 | − | L | COG2925 | exonuclease I | | bll0068 |
| locusC | BJ6T00730 | 61630 | 61998 | − | | | hypothetical protein | | bll0069 |
| locusC | BJ6T00740 | 62148 | 62543 | − | | | hypothetical protein | | bll0070 |
| locusC | BJ6T00750 | 62678 | 62899 | + | | | hypothetical protein | | bsr0071 |
| locusC | BJ6T00760 | 62975 | 64225 | + | | | hypothetical protein | | blr0072 |
| locusC | BJ6T00770 | 64226 | 64681 | + | | | hypothetical protein | | blr0073 |
| locusC | BJ6T00780 | 64674 | 65801 | + | | | hypothetical protein | | blr0074 |
| locusC | BJ6T00790 | 66164 | 66718 | − | | | hypothetical protein | | bll0075 |
| locusC | BJ6T00800 | 66834 | 67748 | − | | | hypothetical protein | | bll0076 |
| locusC | BJ6T00810 | 69844 | 70356 | + | | | hypothetical protein | | blr0077 |
